# Progranulin haploinsufficiency mediates cytoplasmic TDP-43 aggregation with lysosomal abnormalities in human microglia

**DOI:** 10.1186/s12974-024-03039-1

**Published:** 2024-02-13

**Authors:** Wonjae Sung, Min-Young Noh, Minyeop Nahm, Yong Sung Kim, Chang-Seok Ki, Young-Eun Kim, Hee-Jin Kim, Seung Hyun Kim

**Affiliations:** 1 Department of Neurology, College of Medicine, Hanyang University, 222, Wangsimni-Ro, Seongdong-Gu, Seoul, 04763 Republic of Korea; 2 Dementia Research Group, Korea Brain Research Institute, Daegu, Republic of Korea; 3 GC Genome, Yongin, Republic of Korea; 4 Department of Laboratory Medicine, College of Medicine, Hanyang University, Seoul, Republic of Korea

**Keywords:** Frontotemporal dementia, Granulin, Microglia, TDP-43 inclusion, Inflammation

## Abstract

**Background:**

Progranulin (PGRN) haploinsufficiency due to progranulin gene (*GRN*) variants can cause frontotemporal dementia (FTD) with aberrant TAR DNA-binding protein 43 (TDP-43) accumulation. Despite microglial burden with TDP-43-related pathophysiology, direct microglial TDP-43 pathology has not been clarified yet, only emphasized in neuronal pathology. Thus, the objective of this study was to investigate TDP-43 pathology in microglia of patients with PGRN haploinsufficiency.

**Methods:**

To design a human microglial cell model with PGRN haploinsufficiency, monocyte-derived microglia (iMGs) were generated from FTD–*GRN* patients carrying pathogenic or likely pathogenic variants (p.M1? and p.W147*) and three healthy controls.

**Results:**

iMGs from FTD–*GRN* patients with PGRN deficiency exhibited severe neuroinflammation phenotype and failure to maintain their homeostatic molecular signatures, along with impaired phagocytosis. In FTD–*GRN* patients-derived iMGs, significant cytoplasmic TDP-43 aggregation and accumulation of lipid droplets with profound lysosomal abnormalities were observed. These pathomechanisms were mediated by complement C1q activation and upregulation of pro-inflammatory cytokines.

**Conclusions:**

Our study provides considerable cellular and molecular evidence that loss-of-function variants of *GRN* in human microglia can cause microglial dysfunction with abnormal TDP-43 aggregation induced by inflammatory milieu as well as the impaired lysosome. Elucidating the role of microglial TDP-43 pathology in intensifying neuroinflammation in individuals with FTD due to PGRN deficiency and examining consequential effects on microglial dysfunction might yield novel insights into the mechanisms underlying FTD and neurodegenerative disorders.

**Supplementary Information:**

The online version contains supplementary material available at 10.1186/s12974-024-03039-1.

## Background

Frontotemporal lobar degeneration (FTLD) is a clinically and pathologically complex neurodegenerative disorder defined as progressive behavioral abnormality, frontal executive dysfunction, and selective language impairments associated with frontal and anterior temporal lobe degeneration [1, 2]. Frontotemporal dementia (FTD), the most common clinical manifestation of FTLD, has been recognized as a prominent cause of dementia, especially in patients under 65 [1]. Since the first description of the link between a pathogenic variant (PV) of the progranulin gene (*GRN*) and FTD in 2006 [3], more than 70 different pathogenic *GRN* variants in FTD have been reported [3, 4]. *GRN* encodes progranulin (PGRN), a highly conserved, cysteine-rich, secreted glycoprotein [5, 6]. PGRN is involved in many cellular processes, including inflammation, wound healing, tumorigenesis, and neuroprotection [6, 7]. PGRN haploinsufficiency is caused by heterozygous loss-of-function (LOF) mutations of *GRN*, leading to autosomal dominant FTD with TAR DNA-binding protein 43 (TDP-43) positive inclusions in neuron and glial cells [3, 8, 9].

Various studies have demonstrated that PGRN associated with microglia can serve as a critical regulator of inflammation [7, 10–13]. It is well-known that PGRN plays a role in the anti-inflammatory process by reducing pro-inflammatory cytokines and suppressing disease-associated microglial activation, which can lead to neuronal loss [6, 14]. Activated inflammatory response, accumulation of myelin debris in microglial lysosomes, and excessive synaptic pruning via complement activation have been identified in a *Grn* knockout mice model [10–12]. In addition, both global *Grn* knockout mutant mice and microglia-specific *Grn* knockout mutant mice demonstrate extended pro-inflammatory microglial activation and neuronal loss [14]. Likewise, most previous studies have evaluated microglia function in mice models with complete PGRN deficiency. Mice models with heterozygous loss of *Grn* failed to develop gliosis and inflammation. They only exhibited minimal behavior and neuropathologic changes [15–17]. Therefore, microglial function should be identified in human cell models of PGRN haploinsufficiency to investigate the pathology of FTD–*GRN*. In diseases such as Nasu-Hakola disease and hereditary diffuse leukoencephalopathy with spheroids where microglial dysfunction is considered the primary pathomechanism, the term “microgliopathy” has been introduced, emphasizing the pivotal role of microglia [18, 19]. This concept underscores the significance of elucidating pathological mechanisms that give rise to abnormal microglial activation. In the context of FTD–*GRN*, unraveling the pathological phenomena responsible for proinflammatory microglia activation would be crucial for comprehending the disease precisely.

Neuronal and glial cytoplasmic TDP-43 aggregation with a ubiquitinated state is a pathological hallmark of FTD–*GRN*. In FTD–*GRN* cases, it is still unclear how *GRN* dysfunction causes TDP-43 pathology and neurodegeneration. Recent evidence suggests that TDP-43 is involved in neuroinflammatory and immune-mediated mechanisms in FTD pathogenesis [20]. In addition, TDP-43 has relationships with immune and inflammatory pathways, including NF-κB/p65, cGAS/STING, and NLRP3 inflammasome that centers around microglia [20, 21]. Nevertheless, research investigating the presence and mechanisms of TDP-43 pathology in microglia has been scarce.

To investigate whether microglial pathology and dysfunction are present in PGRN haploinsufficiency, we generated monocyte-derived microglial-like cell (iMGs) from two patients diagnosed with FTD–*GRN* (p.M1? and p.W147*). Herein, transcriptional and functional analyses of FTD–*GRN* patient-derived iMGs demonstrated that PGRN deficiency could lead to cytoplasmic TDP-43 deposition with persistent pro-inflammatory environment by microglia activation, dysregulation of lysosomal function, and altered lipid metabolism. This study also suggests new evidence for the relationship between TDP-43 aggregation and microglia-mediated excessive inflammatory reactions, elucidating the underlying mechanism of TDP-43 proteinopathy in FTD–*GRN*. These pathological and functional abnormalities found in human microglia harboring PGRN haploinsufficiency could provide crucial insight into the development of therapeutic strategies for FTD–*GRN.*

## Materials and methods

### Clinical and genetic characteristics of FTD–GRN subjects and genetic analyses

Two patients diagnosed with FTD–*GRN* were included in this study. Blood, cerebrospinal fluid (CSF), and skin samples were obtained from these patients and three healthy controls. Three controls were recruited from sex- and age-segregated healthy individuals (control-1, a 55-year-old male; control-2, a 63-year-old female; and control-3, a 75-year-old male). Demographic and clinical characteristics of FTD–*GRN* patients and healthy controls are summarized in Table S1. Clinical diagnosis of FTD was made according to current consensus criteria [22, 23]. *GRN* variants were identified by Sanger sequencing and whole-exome sequencing. A Wizard Genomic DNA Purification Kit (Promega, Madison, WI, USA) was used to extract genomic DNAs from peripheral blood leukocytes according to the manufacturer’s instructions. Whole-exome sequencing libraries were generated utilizing an Agilent SureSelect All Exon 50 Mb Kit (Agilent, Santa Clara, CA, USA) according to the manufacturer’s instructions. The flow cell was loaded onto a NextSeq 500 sequencing system (Illumina Inc., San Diego, CA, USA) for sequencing with 2 × 100 bp read lengths. Reads were mapped to the GRCh37/hg19 build using the Burrows-Wheeler Aligner. Variants were named using GATK software. All variants with allele frequencies > 0.01 were filtered out based on various public databases, including the genome aggregation database (gnomAD, https://gnomad.broadinstitute.org) and the Korean Reference Genome Database (KRGDB, http://coda.nih.go.kr/coda/KRGDB/index.jsp). All identified variants were classified according to the American College of Medical Genetics and Genomics and the Association for Molecular Pathology (ACMG/AMP) guidelines [24] and ClinGen recommendations (https://clinicalgenome.org/working-groups/sequence-variant-interpretation/). The study protocol was approved by the Institutional Review Board (IRB) of Hanyang University Hospital (HYUH 2017-01-043-002). Written informed consent was obtained from all patients involved in the study.

### Generation of human microglia-like cells (iMGs) from PBMCs

To generate iMGs from patients and controls, peripheral blood mononuclear cells (PBMCs) from whole blood were used to differentiate monocytes into microglia-like cells according to a previously published method [25]. Briefly, PBMCs were isolated by density gradient centrifugation using Ficoll (GE Healthcare, Uppsala, Sweden) and resuspended in RPMI-1640 (Gibco, Grand Island, NY, USA) supplemented with 10% fetal bovine serum (FBS; Gibco) and 1% antibiotic/antimycotic (Invitrogen, Carlsbad, CA, USA) and incubated at 37 °C overnight with 5% CO_2_. The next day, adherent cells (monocytes) were cultured in RPMI-1640 Glutamax (Gibco) supplemented with 1% antibiotic/antimycotic, recombinant granulocyte–macrophage colony-stimulating factor (GM-CSF) (R&D Systems, Minneapolis, MN, USA), and recombinant interleukin (IL)-34 (IL-34) (R&D Systems) for 14 days to cultivate iMGs cells. To generate fibroblasts from identical FTD–*GRN* patient, adult human fibroblasts were extracted from forearm skin by punch biopsy and cultured at 37 °C with 5% CO_2_ in Dulbecco’s modified Eagles’ medium (DMEM) supplemented with non-essential amino acids (Gibco), sodium bicarbonate (Sigma-Aldrich), 1% (vol/vol) penicillin/streptomycin/Fungizone (Cellgro), and 20% FBS.

### Enzyme‑linked immunosorbent assay (ELISA) for PGRN, TREM2, NfL, and C1q

To measure PGRN, TREM2, NfL, and complement C1q levels, blood samples were collected into ethylenediamine tetra-acetic acid (EDTA) tubes and CSF samples were collected into polypropylene tubes by lumbar puncture. Samples were centrifuged at 3500 rpm for 20 min at 4 °C, aliquoted, and then stored at − 80 °C until use. To isolate cell culture-conditioned medium, fresh culture medium was added to cells at 24 h before collection and centrifuged at 12,000 rpm for 10 min at 4 °C to remove cellular debris. Concentrations of PGRN from plasma and secreted PGRN from culture media of iMGs were determined using a human PGRN enzyme-linked immunosorbent assay (ELISA) kit (Adipogen, Coger S.A.S., France) according to the manufacturer’s instructions. CSF neurofilament light chain (NfL) was measured using an ELISA kit (UmanDiagnostics AB, Umeå, Sweden). Soluble TREM2 (sTREM2) was measured using a Human TREM2 SimpleStep ELISA kit (Abcam, ab 224881) according to the manufacturer’s instructions. Complement C1q proteins in each iMGs CM were measured using a Human Complement C1q ELISA kit (Abcam, ab170246, Boston, MA, USA) according to the manufacturer’s instructions. All ELISAs were performed by experienced technicians who were blinded to basic information of patients. All samples and standards were measured in triplicate. Means of duplicate experiments were used for statistical analyses.

### Quantitative real-time PCR analysis of iMGs gene expression

Gene expression in iMGs was measured by quantitative real-time polymerase chain reaction (PCR) analysis as described previously [26]. Total RNA was extracted using TRIzol Reagent (Life Technologies, Carlsbad, USA) and reverse transcribed using a High-Capacity cDNA Reverse Transcription kit (Applied Biosystems, Waltham, MA, USA). qPCR was performed using an SYBR Green PCR Master Mix (Applied Biosystems) and primers shown below. Data were normalized to GAPDH expression level. All primers were designed using GenScript primer design software.

**Table:** 

Gene	Forward (5ʹto 3ʹ)	Reverse (5ʹ to 3ʹ)
*GRN*	GGGCCTCATTGACTCCAAG	GTGGTGTAAGCGGTACCCTC
*P2RY12*	TTCAAACCCTCCAGAATCAACAG	GTGCACAGACTGGTGTTACC
*TMEM119*	CCACTCTCGCTCCATTCG	CAGCAACAGAAGGATGAGGA
*TGFBR1*	CACAGAGTGGGAACAAAAAGGT	CCAATGGAACATCGTCGAGCA
*CXCR1*	TGGGGCCTTCACCATGGAT	GCCAATGGCAAAGATGACGGAG
*IL-1β*	ACAGATGAAGTGCTCCTTCCA	GTCGGAGATTCGTAGCTGGAT
*TNF-α*	GGAGAAGGGTGACCGACTCA	CTGCCCAGACTCGGCAA
*IL-6*	ACTCACCTCTTCAGAACGAATTG	CCATCTTTGGAAGGTTCAGGTTG
*LAMP1*	ACGTTACAGCGTCCAGCTCAT	TCTTTGGAGCTCGCATTGG
*LAMP2*	TGCTGGCTACCATGGGGCTG	GCAGCTGCCTGTGGAGTGAGT
*CTSB*	CAGCGTCTCCAATAGCGA	AGCCCAGGATGCGGAT
*CTSD*	GCAAACTGCTGGACATCGCTTG	GCCATAGTGGATGTCAAACGAGG
*ATP6AP2*	AGGCAGTGTCATTTCGTACC	GCCTTCCCTACCATATACACTC
*LGALS3*	ATGGCAGACAATTTTTCGCTCC	GCCTGTCCAGGATAAGCCC
*PLIN3*	TATGCCTCCACCAAGGAGAG	ATTCGCTGGCTGATGCAATCT
*GAPDH*	GGTATGACAACGAATTTGGC	GAGCACAGGGTACTTTATTG

### Comprehensive immunocytochemical analysis of microglial cells

For analysis of protein expression by immunostaining, cultured cells were fixed with 4% formaldehyde in phosphate-buffered saline (PBS) for 20 min at room temperature (RT), permeabilized with 0.2% Triton X-100 in PBS at RT for 15 min, and blocked with 1% bovine serum albumin (BSA) in PBS at RT for one hour. Cells were then incubated with primary antibodies overnight at 4 °C and labeled with secondary antibodies for 60 min at RT, followed by counterstaining with a nuclear marker 4′,6-diamidino-2-phenylindole (DAPI) (Sigma-Aldrich, D9542). The following primary antibodies were used: anti-ionized calcium-binding adaptor protein 1 (IBA1, 1:1000; ab5076, RRID:AB_2224402, Abcam), anti-IBA1 (1:200; 019-19741, RRID:AB_839504, Wako Chemicals, Richmond, VA, USA), anti-progranulin (1:1000; AF2420, RRID:AB_2114489, R&D Systems), anti-lysosomal-associated membrane protein 1 (LAMP1, 1:500; ab25630, RRID:AB_470708, Abcam), anti-transmembrane protein 119 (TMEM119, 1:200; ab185337, RRID:AB_2921338, Abcam), anti-cluster differentiation 68 (CD68,1:200; ab201340, RRID:AB_2920880, Abcam), anti-TDP-43 (1:200; 10782-2AP, RRID:AB_615042, Proteintech, Rosemont, IL, USA), anti-pTDP-43 (S409/410) (1:100; TIP-PTD-M01, RRID: AB_3083546, Cosmo Bio, Carlsbad, CA, USA), anti-ubiquitin (1:500; NB300-129, RRID:AB_2180545, Novus Bio, Centennial, CO, USA), and anti-transcription factor EB (TFEB, 1:100; sc-166736, RRID:AB_2255943, Santa Cruz Biotechnology, Dallas, Texas, USA). Secondary antibodies included Alexa Fluor 488, 555, and 633-conjugated antibodies (1:500; A11001, RRID:AB_3083547, A11008, RRID:AB_143165, A21422, RRID:AB_2535844, A21428, RRID:AB_2535849, and A21082, RRID:AB_10562400; Invitrogen). Images were acquired with a Leica TCS SP8 laser-scanning confocal microscope using an HC PL APO CS2 63x/1.40 objective. To assess cytoplasmic TDP-43 aggregation, the percentage of cytoplasmic TDP-43 immunoreactivity in each defined area was quantified as described previously [27]. A threshold was defined for background correction to calculate the percentage of cytoplasmic TDP-43 immunoreactivity. ImageJ software (National Institute of Health, USA) was used to measure the pixel rate in the area above the threshold of TDP-43 labeling. In addition, the mean ratio of cells with TDP-43 positivity within their cytoplasm was calculated using analyzed confocal images by counting the number of intracytoplasmic TDP-43 positive cells compared to the number of IBA1 positive cells. For quantitative analysis of intracellular levels of CD68 and LAMP1 in microglia culture, the entire cell body was selected and the fluorescence intensity was measured directly using ImageJ after a threshold application. To quantify nuclear to cytoplasmic TFEB ratio, the nucleus and the entire cell were selected and the fluorescence intensity was measured directly with ImageJ. Intensity from the entire cell was subtracted from nuclear to calculate cytoplasmic TFEB intensity. For each experiment, at least 10 pairs of cells were measured. Data from five independent experiments were used for statistical analysis.

### Assessment of microglial functions

To examine microglial phagocytic capacity, iMGs were incubated with red fluorescent microspheres (L3030, Sigma-Aldrich) for 2 h at 37 °C, washed with PBS three times to remove fluorescent micro-spheres not phagocytized, fixed, and stained with Alexa Fluor 488 phalloidin (1: 1,000; Molecular Probes, Eugene, OR, USA) according to the manufacturer’s protocol. Images were acquired using a confocal microscope. The number of phagocytized beads was counted using ImageJ software.

### Western blotting of iMGs and fibroblasts

After iMGs and fibroblast cells were washed twice with PBS, they were incubated on ice in radioimmunoprecipitation assay buffer (RIPA buffer) containing proteinase and phosphatase inhibitors for 10 min to isolate an RIPA-soluble fraction. Insoluble pellets were washed with RIPA buffer and extracted twice with urea buffer (7 M urea, 2 M thiourea, 4% CHAPS, and 30 mM Tris, pH 8.5). After sonication, samples were centrifuged at 200,000 g for 1 h at 24 °C and the supernatant was collected as an RIPA-insoluble fraction (urea-soluble). Protein concentrations were determined by the Bradford assay, a colorimetric protein assay, and then standardized. Equal amounts of protein were analyzed by Western blotting using indicated antibodies. Primary antibodies included anti-progranulin (PGRN, 1:1,000; AF2420, RRID:AB_2114489, R&D), anti-TDP-43 (1:1000; 10782-2AP, RRID:AB_615042, Proteintech), anti-pTDP-43 (S409/410) (1:1000; TIP-PTD-M01, RRID:AB_3083546, Cosmo Bio), anti-p62 (1:1,000; ab109012, RRID:AB_2810880, Abcam), anti-ubiquitin (1:5000; NB300-129, RRID:AB_2180545, Novus bio), anti-sortilin 1(SORT1, 1:1000; ab16640, RRID:AB_2192606, Abcam), anti-transmembrane protein 106B (TMEM106B, 1:1000; 20995–1-AP, RRID:AB_10694293, Proteintech), and anti-GAPDH (1:1,000; sc-25778, RRID:AB_10167668, Santa Cruz). The membrane was then probed with horseradish peroxidase-conjugated secondary antibodies (Santa Cruz). Immunoreactive bands were visualized using a West-Q Chemiluminescent Substrate Plus Kit (GenDEPOT, Barker, TX, USA). Membranes were then re-probed for GAPDH as an internal control. This experiment was performed by collecting 50 ml of blood three times from the same patient and generating it into iMGs. However, some Western blot experiments could not be repeated due to insufficient cell protein, and only one blot result was presented in the paper. The results obtained in this way, Figs. 3D and 4H, could not be plotted by quantitative analysis.

### Complement treatment in microglial cells

To determine effects of complement C1q in microglial cells, murine microglial BV2 cells were maintained in DMEM (Life Technologies) supplemented with 10% FBS and 1% penicillin/streptomycin under standard culture conditions (95% relative humidity with 5% CO_2_ at 37 °C). Adherent cells were split using 1 × TrypLE (Gibco). BV2 cells were seeded at a density of 5 × 10^4^ cells per coverslip on poly-l-lysine coated glass coverslips in DMEM + 10% FBS and treated with 1 μg/ml purified human complement C1q (Sigma) for 24 h. To knockdown (KD) the *GRN* gene in microglial cells, pre-designed Silencer® human *GRN* siRNA and control siRNA (Invitrogen) were transfected into BV2 cells using RNAiMAX (Invitrogen) according to the manufacturer’s protocol.

### Lipid droplet analysis in microglial cells

To detect lipid droplet formation, iMGs were immunostained with IBA1, incubated in PBS with BODIPY 493/503 (4,4-difluoro-1,3,5,7,8-pentamethyl-4-bora-3a,4a-diaza-s-indacene) (1:1,000 from a 1 mg/mL stock solution in DMSO; D3922, Thermo Fisher Scientific) as a lipid droplet marker for 10 min at RT, washed three times in PBS, and counterstained with DAPI. The percentage of lipid-droplet-containing iMGs was determined by calculating the rate of BODIPY^+^ IBA1^+^ cells after counting the total number of IBA1^+^ cells and IBA1^+^ cells with BODIPY^+^ lipid droplets. In addition, BODIPY^+^ fluorescence intensity per cell was analyzed using ImageJ software to determine the relative concentration of lipid droplets.

Following specific treatments, BV2 cells were fixed in 4% PFA for 30 min, washed three times in PBS, and incubated in PBS with BODIPY 493/503 (1:1000) for 10 min at RT. Cells were then washed with PBS three times and counterstained with DAPI. Percentages of lipid-droplet-containing BV2 cells in total cells and BODIPY^+^ cells were analyzed using ImageJ software. The average size of lipid droplets in the BODIPY^+^ signal was analyzed using the ‘analyze particles’ function of ImageJ software.

### Statistical analysis

Comparisons were performed using unpaired *t* tests or one-way analysis of variance (ANOVA) with post-hoc Tukey tests using GraphPad Prism 9 software. Data are presented as mean ± standard error of mean. Statistical significance was considered when *p* values were less than 0.05. Significance was indicated as follows: *, *p* < 0.05; **, *p* < 0.01; ***, *p* < 0.001; and ****, *p* < 0.0001.

## Results

### Clinical and genetic characteristics of patients diagnosed with FTD–GRN

Two different *GRN* variants were identified, one with a recurrent pathogenic variant (c.1A>G, p.M1?) and another with a novel, likely pathogenic variant (c.441G>A, p.W147*). The p.M1? variant is known to affect the initiation codon residue. Meanwhile, the de novo *GRN* variant p.W147* located in exon 5 of *GRN* is predicted to generate a premature stop codon. The patient carrying the p.M1? variant was classified as familial FTD–*GRN* having mixed FTD presenting with behavior variant FTD and semantic variant primary progressive aphasia (svPPA). The other patient was diagnosed with simplex FTD, presenting with mild svPPA. Clinical and genetic characteristics of these FTD–*GRN* patients and controls are summarized in Additional file 1: Table S1 and Figures S1 and S2.

### PGRN haploinsufficiency causes inflammatory phenotype and defective phagocytosis in microglia

**Fig. 1:**
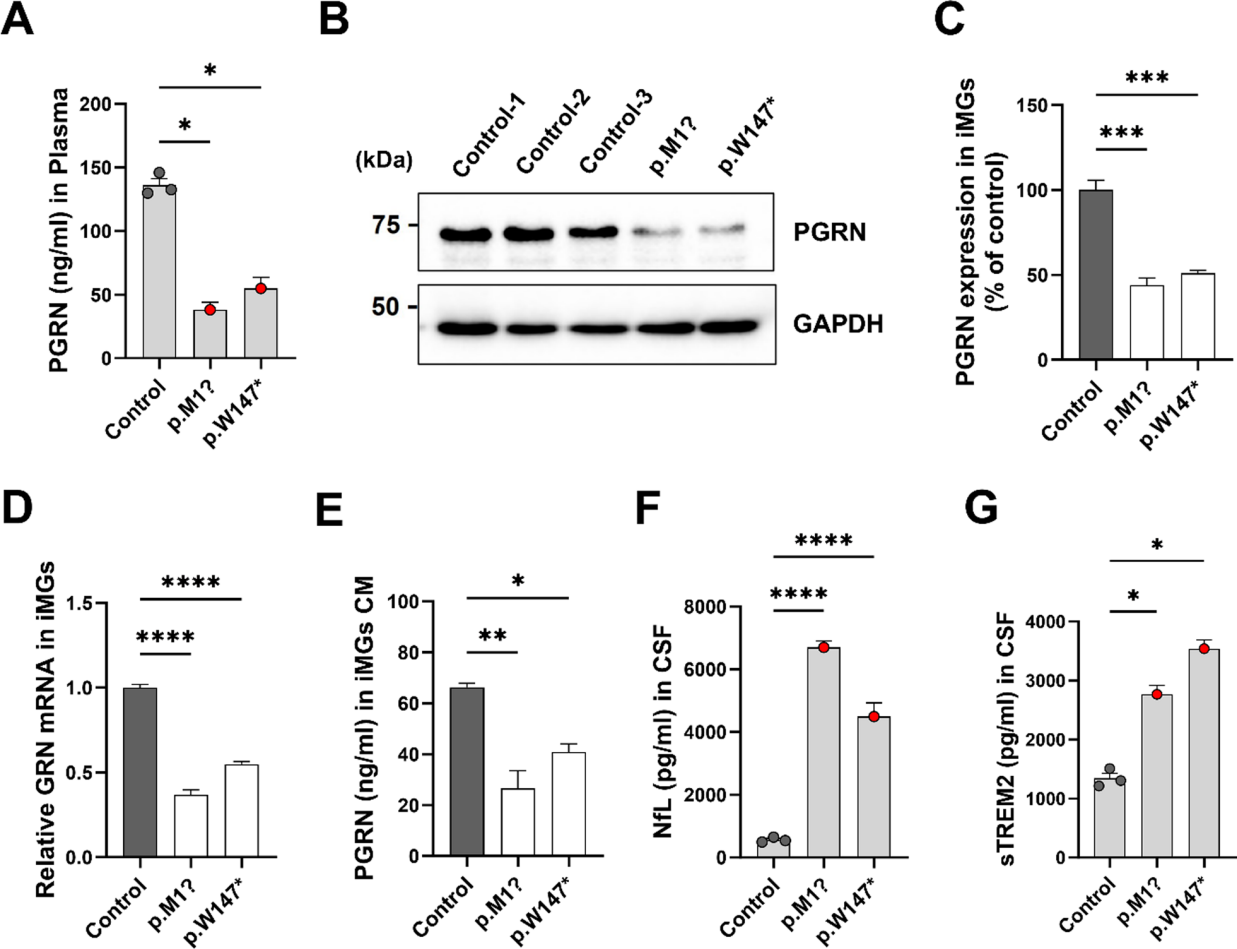
FTD–*GRN* patients-derived plasma and iMGs show reduced PGRN levels. **A** Human PGRN levels in FTD–*GRN* patients’ plasma vs. controls (*n* = 3) measured by ELISA, mean ± SEM; *p < 0.05 (one-way ANOVA, Tukey’s test). **B** Western blot of PGRN in iMGs cell lysates, Glyceraldehyde-3-phosphate dehydrogenase (GAPDH) as loading control. **C** Quantified PGRN protein normalized to controls (*n* = 3), mean ± SEM; ***p < 0.001 (one-way ANOVA, Tukey’s test). **D** qPCR of *GRN* mRNA in FTD–*GRN* vs. control iMGs, normalized to control, mean ± SEM; ****p < 0.0001 (one-way ANOVA, Tukey’s test). **E** Secreted PGRN in conditioned media (CM) of FTD–*GRN* vs. control iMGs by ELISA, mean ± SEM; *p < 0.05, **p < 0.01. F. NfL levels in CSF of FTD–*GRN* patients vs. controls (*n* = 3), one-way ANOVA, Tukey’s test. **G** Soluble TREM2 (sTREM2) in CSF of FTD–*GRN* vs. controls (*n* = 3), mean ± SEM; *p < 0.05, ****p < 0.0001 (one-way ANOVA, Tukey’s test)

To understand the contribution of PGRN haploinsufficiency to microglial dysfunction in FTD–*GRN*, we generated monocyte-derived microglia-like cells (iMGs) from blood as previously described [25]. It is known that iMGs generated by this method can recapitulate disease-related phenotypes in neurodegenerative diseases as a suitable model for studying human microglia. It has been developed over the past decade and used to study the functional role of disease-related genetic variants [28–30]. First, plasma PGRN levels in the two FTD–*GRN* patients and three controls were analyzed to identify characteristics of PGRN haploinsufficiency in *GRN* variants. Plasma PGRN levels were significantly reduced in *GRN* variant carriers compared to those in controls (Fig. 1A). We examined expression levels of *GRN* mRNA and PGRN protein in iMGs from two FTD–*GRN* patients and three controls to determine possible differences in PGRN expression between them. PGRN protein expression and *GRN* transcript levels were reduced by more than 50% in iMGs from FTD–*GRN* patients compared to those in controls (Fig. 1B, D). Amounts of secreted PGRN were also reduced in culture media (CM) of iMGs from FTD–*GRN* patients compared to those in controls based on ELISA (Fig. 1E). These results indicated that this study successfully established a patient-specific human microglia cell model of PGRN haploinsufficiency. To elucidate and reinforce the evidence of neurodegeneration and inflammation in FTD–*GRN* patients, we analyzed levels of neurofilament light chain (NfL), a biomarker of neurodegeneration [31], and soluble TREM2 (sTREM2), a biomarker of microglial activation [32, 33], in CSF samples of FTD–*GRN* patients and controls. CSF levels of NfL and sTREM2 were significantly increased in FTD–*GRN* patients compared to those in controls (Fig. 1F, G).

**Fig. 2:**
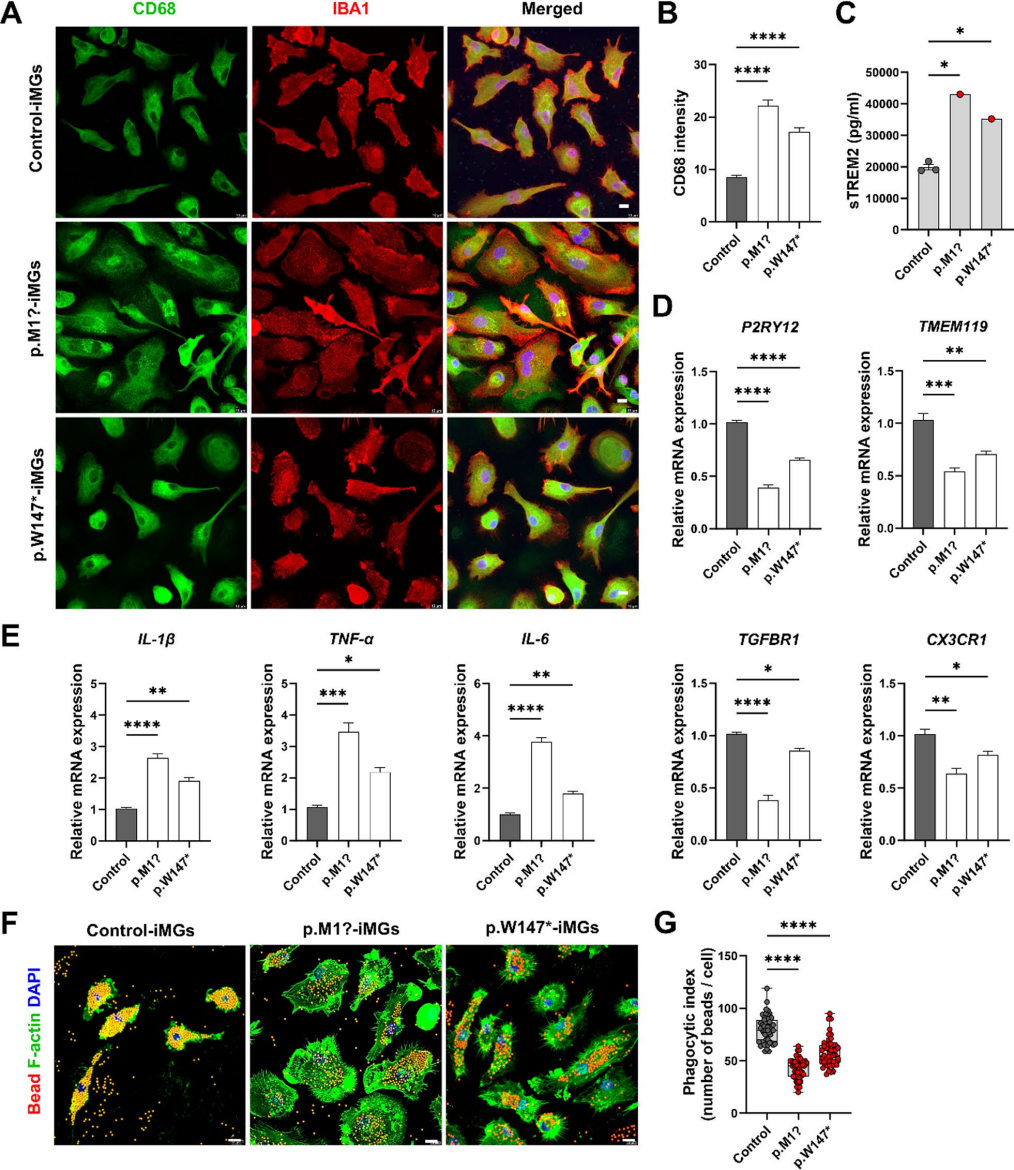
Patient-derived iMGs carrying *GRN* variants represent an activated inflammation state and defective phagocytosis. **A** Fluorescence images of CD68 (green, reactive microglia) and Ionized calcium-binding adapter molecule 1 (IBA1, red, microglia) in FTD–*GRN* patient-derived iMGs vs. controls. Nuclei stained with DAPI; scale bar at 10 µm. **B** Quantification of CD68 intensity in IBA1^+^ iMGs from over 50 cells/experiment in three independent experiments, presented as mean ± SEM. Statistical analysis: ****p < 0.0001, one-way ANOVA, Tukey’s test. **C** ELISA quantification of sTREM2 in iMGs’ conditioned media (*n* = 3), shown as means ± SEM; *p < 0.05; one-way ANOVA, Tukey’s test). **D** Relative mRNA levels of microglia genes (*P2RY12*, *TMEM119*, *TGFBR1*, *CX3CR1*) in iMGs, normalized to control. Data from three experiments, mean ± SEM; *p < 0.05, **p < 0.01, ***p < 0.001, and ****p < 0.0001; one way ANOVA with post hoc Tukey’s test. **E** mRNA of pro-inflammatory genes (*IL-1β*, *TNF-α*, *IL-6*) in iMGs, normalized to control. Three experiments, mean ± SEM; *p < 0.05, **p < 0.01, ***p < 0.001, and ****p < 0.0001; one way ANOVA with post hoc Tukey’s test. **F** Fluorescence images of bead phagocytosis in iMGs; F-actin (green), nuclei (DAPI). Scale bar, 10 µm. **G** Quantification of phagocytosed beads/iMG cell, over 50 cells/experiment from three independent experiments, mean ± SEM; ****p < 0.0001(ANOVA with post hoc Tukey’s test)

Expression levels of activated microglial marker CD68 and microglial marker IBA1 were evaluated by immunostaining to explore PGRN-dependent microglial state in FTD–*GRN* patient-derived iMGs. FTD–*GRN* patient-derived iMGs showed enhanced CD68 immunoreactivity compared to control-derived iMGs (Fig. 2A, B). In addition, most microglial cells in the patient carrying the p.M1? variant showed morphological changes to an amoeboid form with a larger soma (Fig. 2A), a characteristic feature of activated microglia [34]. Furthermore, sTREM2 levels in CM from control and FTD–*GRN* patient-derived iMGs were analyzed to assess microglial activation. The media from iMGs of FTD–*GRN* patients showed elevated levels of sTREM2 compared to controls (Fig. 2C). Next, mRNA expression levels of homeostatic microglial and inflammation-related genes were determined using qPCR to determine changes in gene expression in iMGs derived from patients with FTD–*GRN*. Compared to control-derived iMGs, FTD–*GRN* patient-derived iMGs showed decreased expression levels of homeostatic microglia-specific genes (*P2RY12*, *TMEM119*, *TGFBR1*, and *CX3CR1*) but increased expression levels of inflammation-related genes (*IL-1β*, *TNF-α*, and *IL-6*) (Fig. 2D, E). Furthermore, phagocytic function of red fluorescent bead was significantly reduced in iMGs from FTD–*GRN* patients compared to that in control iMGs (Fig. 2F, G). These findings support that loss of PGRN in human iMGs can lead to their pro-inflammatory state, failing to maintain their homeostatic molecular signatures and impaired phagocytosis capacity due to exacerbated neuroinflammation.

### PGRN haploinsufficiency induces cytoplasmic TDP‑43 accumulation with complement activation in microglia

**Fig. 3:**
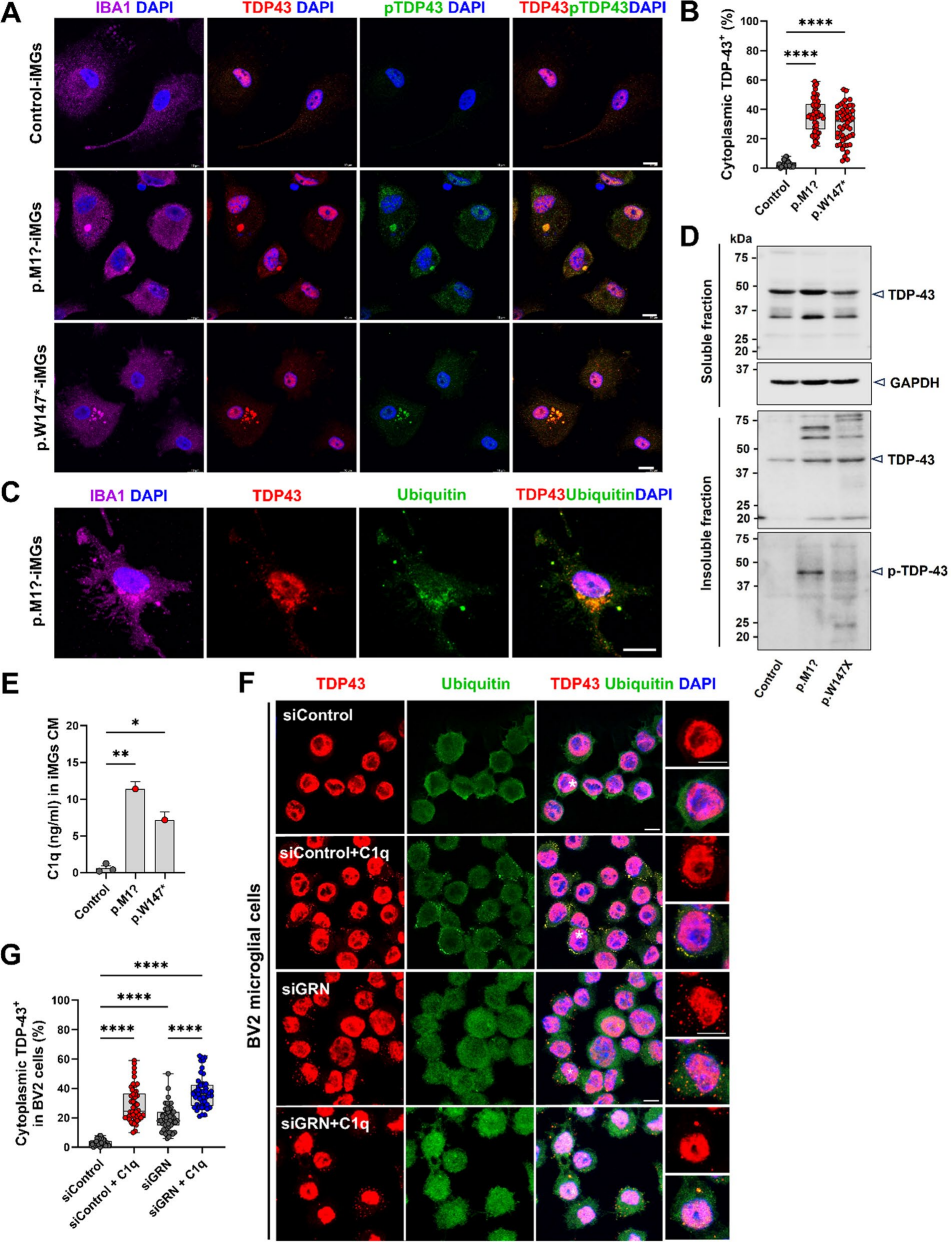
Patient-derived iMGs carrying *GRN* variants display abnormal TDP-43 aggregation. **A** Fluorescence images of IBA1 (purple, microglia), TDP-43 (red), pTDP-43 (Ser409/410, green) in FTD–*GRN* iMGs vs. controls; DAPI for nuclei. Scale bar, 10 µm. **B** Cytoplasmic TDP-43 in IBA1^+^ iMGs quantified, 50 + cells/subject, three experiments. Mean ± SEM; ****p < 0.0001 (one-way ANOVA, Tukey’s test). **C** Images of IBA1 (purple), TDP-43 (red), ubiquitin (green) in FTD–*GRN* iMGs. DAPI-stained nuclei. Scale, 10 µm. **D** Western blot for total/phosphorylated TDP-43 in FTD–*GRN* vs. control iMGs; GAPDH for soluble fraction. **E** Complement C1q concentration in CM by ELISA (*n* = 3); *p < 0.05, **p < 0.01 (one-way ANOVA with post hoc Tukey’s test). **F** TDP-43 and ubiquitin in BV2 cells post-siRNA *GRN* knockdown, with/without 1 μg/ml C1q for 24 h. DAPI nuclei. Scale, 10 µm. G. Quantification of cytoplasmic TDP-43 granules in condition (**F**), 50+ cells/condition, 3 experiments. Mean ± SEM; ****p < 0.0001

Previous reports have demonstrated that PGRN depletion can induce cytosolic TDP-43 accumulation in several cell models [35, 36]. However, direct microglial TDP-43 pathology has not yet been demonstrated yet. We examined TDP-43 immunoreactivity in control and FTD–*GRN* patient-derived iMGs to determine whether PGRN depletion in the human microglial-like cell model might represent critical aspects of FTD–TDP pathophysiology. In control-derived iMGs, we observed TDP-43 signal exclusively in nuclei of IBA1^+^ cells. The TDP-43 signal in FTD–*GRN* patient-derived iMGs was presented in nuclear and cytoplasmic TDP-43^+^ condensates. Cytoplasmic TDP-43 inclusions showed a granular, dot-like, and round form (Fig. 3A). These findings were similar to neuronal TDP-43 proteinopathy in patients with FTD [37, 38]. Notably, the percentage of cytoplasmic TDP-43 positive cells was significantly increased in FTD–*GRN* patient-derived iMGs compared to control-iMGs (Fig. 3B). Cytoplasmic TDP-43 inclusions were positive for a pathological form of phosphorylated TDP-43 (pTDP-43 at Ser409/410) and colocalized with ubiquitin (Fig. 3A, C). To further confirm these results, cell lysates from control and FTD–*GRN* patient-derived iMGs were separated into soluble and insoluble fractions and analyzed by western blots. We found that levels of insoluble TDP-43 were elevated in FTD–*GRN* patient-derived iMGs compared to those in the control. Only FTD–*GRN* patient-derived iMGs exhibited TDP-43 phosphorylation at S409/410 residues detectable in insoluble fractions (Fig. 3D).

*GRN* mutant FTD patients are known to have excessive complement production [10]. It has recently been reported that complement released from microglia of Grn^−/−^ mice can promote neuronal TDP-43 proteinopathy [39]. To determine whether *GRN*–LOF microglia could facilitate complement production, complement C1q levels were analyzed in media from control and FTD–*GRN* patient-derived iMGs. ELISA revealed that complement C1q was significantly increased in iMGs CM from FTD–*GRN* patients compared to that in iMGs CM from controls (Fig. 3E). We next investigated whether complement C1q was sufficient to induce TDP-43 proteinopathy in microglia and whether this phenomenon was affected by *GRN* loss. BV2 microglial cells were transfected with *GRN* siRNA, treated with human complement C1q for 24 h, and stained with TDP-43 antibody. As a result, complement C1q treatment generated cytoplasmic TDP-43 granules colocalized with ubiquitin in BV2 microglial cells. Furthermore, under complement C1q treatment conditions after *GRN* KD using siRNA, the production of cytoplasmic TDP-43 granules was induced and some larger granules were observed (Fig. 3F, G). These findings suggest that *GRN*–LOF can activate complement C1q in patient-derived microglial cells, resulting in a neurotoxic inflammatory state. TDP-43 condensates may transiently form by inflammatory milieu with complement activation in microglia. PGRN deficiency impaired the ability of microglia to clear cytoplasmic TDP-43 condensates, leading to a cascade that locked microglial cells into their pro-inflammatory state and prevented them from transitioning back to homeostasis. Thus, chronic inflammatory conditions could be related to the production of cytoplasmic TDP-43 aggregation in patient-specific microglia, which can result in disease mechanisms linked to FTD.

### PGRN haploinsufficiency leads to dysregulation of lysosomal markers and abnormal lipid droplet formation in microglia

**Fig. 4:**
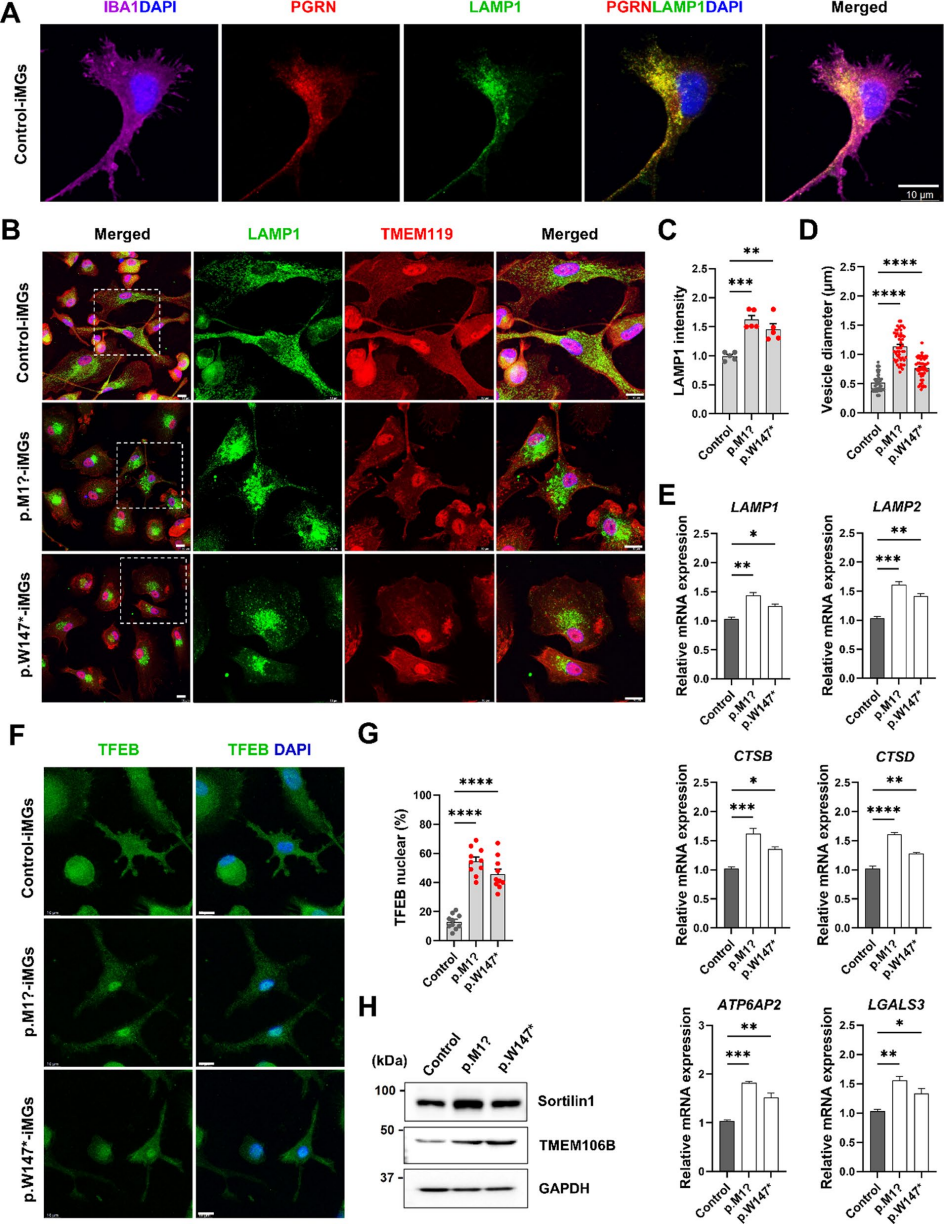
Patient-derived iMGs carrying *GRN* variants exhibit enlarged lysosomal abnormalities. **A** Fluorescence images of PGRN (red), Lysosome-associated membrane protein 1 (LAMP1, green), and IBA1 (purple) in control iMGs; DAPI-stained nuclei. Scale, 10 µm. **B** Images of LAMP1 and Transmembrane Protein 119 (TMEM119) in FTD–*GRN* and control iMGs; higher magnification of selected areas. DAPI nuclei. Scale, 10 µm. **C** LAMP1 intensity quantified in TMEM119^+^ iMGs, 50 + cells/subject, 5 experiments; mean ± SEM, **p < 0.01, ***p < 0.001 (one-way ANOVA with post hoc Tukey’s test). **D** LAMP1^+^ vesicle size quantification in iMGs, 50 + cells/subject, 3 experiments; mean ± SEM, ***p < 0.001, ****p < 0.0001 (one-way ANOVA with post hoc Tukey’s test). **E** mRNA of lysosome-related genes (*LAMP1*, *LAMP2*, *CTSB*, *CTSD, ATP6AP2*, and *LGALS3*) in FTD–*GRN* vs. control iMGs, normalized to control; mean ± SEM, *p < 0.05, **p < 0.01, ***p < 0.001, and ****p < 0.0001 (one-way ANOVA with post hoc Tukey’s test). **F** TFEB fluorescence in FTD–*GRN* and control iMGs; DAPI nuclei. Scale, 10 µm. **G** Nuclear TFEB cell count in iMGs (*n* = 50/subject); mean ± SEM, ****p < 0.0001 (one-way ANOVA with post hoc Tukey’s test). **H** Western blot for Sortilin1 (SORT1), TMEM106B, GAPDH in FTD–*GRN* and control iMGs; GAPDH as loading control

Homozygous *GRN* variants can cause neuronal ceroid lipofuscinosis, a lysosomal storage disorder, suggesting that PGRN plays an essential role in lysosomal homeostasis [40]. Immunostaining analysis was performed to examine microglial subcellular distribution of endogenous PGRN using control-derived iMGs. We found that PGRN was expressed in iMGs (IBA1^+^ microglia). It was localized in lysosomal compartments, showing colocalization with lysosomal-associated membrane protein 1 (LAMP1), a lysosome marker (Fig. 4A). We immunostained with antibodies against LAMP1, a lysosome membrane protein, and TMEM119 as a homeostatic microglia marker in FTD–*GRN* and control-derived iMGs to corroborate the effect of PGRN loss on lysosome in microglia. LAMP1 intensity detected in FTD–*GRN* patient-derived iMGs was higher than that in control-iMGs and LAMP1-positive lysosomes were markedly enlarged (Fig. 4B–D). In addition, mRNA levels of lysosome-related genes, including lysosomal membrane protein (*LAMP1* and *LAMP2*), lysosomal proteinase cathepsins (*CTSB* and *CTSD*), lysosomal acidification (ATPase H + transporting lysosomal accessory protein 2, *ATP6AP2*), and damaged lysosomes (*LGALS3*, gene coding Gal-3) were significantly increased in iMGs from FTD–*GRN* patients compared to those in control-derived iMGs (Fig. 4E).

Since enlarged lysosomes and activation of lysosome-related genes were observed in iMGs from FTD–*GRN* patients, we investigated lysosomal damage response caused by PGRN loss mediated by transcription factor EB (TFEB), the master regulator of lysosomal biogenesis and function [41]. Subcellular localization of TFEB in iMGs was assessed to clarify the activation of TFEB in lysosomal damage by *GRN* variants. FTD–*GRN* patient-derived iMGs showed increased TFEB nuclear translocation compared to control-derived iMGs (Fig. 4F, G). Whether the loss of PGRN affected the expression of lysosome-related proteins, including sortilin (SORT1), a clearance receptor of PGRN, and transmembrane protein 106 B (TMEM106B), a lysosomal membrane protein previously implicated as a genetic risk factor for FTLD–TDP [42], was additionally investigated. Patient iMGs carrying *GRN*–LOF variants show increased expression of SORT1 and TMEM106B compared to control iMGs (Fig. 4H), consistent with increased TMEM106B in brains of patients diagnosed with FTD [43].

**Fig. 5:**
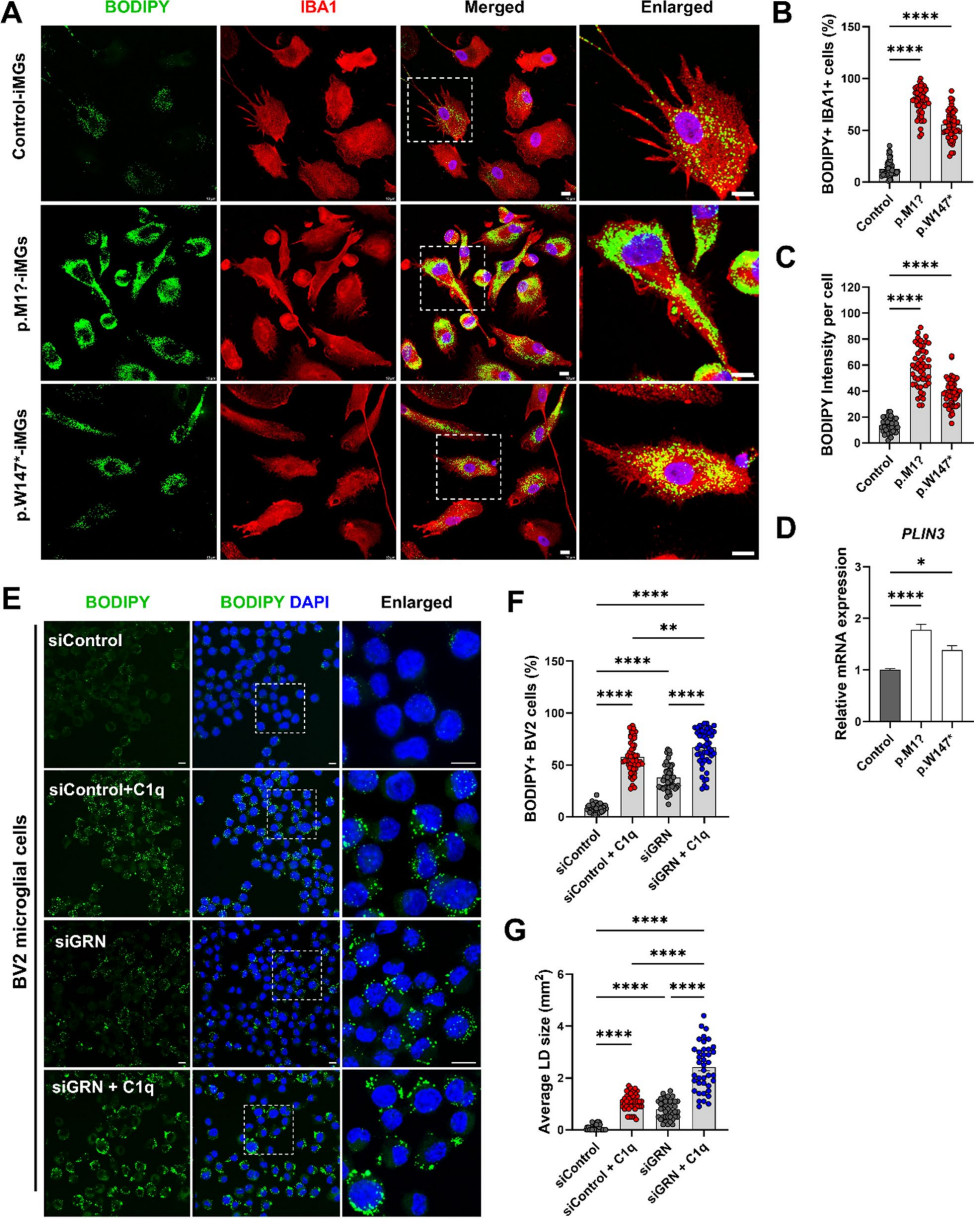
Patient-derived iMGs carrying *GRN* variants induce aberrant lipid droplet formation. **A** Fluorescence images of BODIPY (lipid droplets, green) and IBA1 (microglia, red) in FTD–*GRN* iMGs vs. controls; DAPI-stained nuclei. Scale, 10 µm. **B** Percentage of BODIPY^+^ IBA1^+^ cells quantified, 50 + cells/experiment, 3 experiments; mean ± SEM, ****p < 0.0001 (one-way ANOVA, Tukey’s test). **C** BODIPY intensity/cell, 50 + cells/experiment, 3 experiments; mean ± SEM, ****p < 0.0001 (one-way ANOVA, Tukey’s test). **D** Relative mRNA of *Perilipin 3* (*PLIN3*) in FTD–*GRN* vs. control iMGs, normalized to control; mean ± SEM, **p < 0.01, ****p < 0.0001. **E** BODIPY in BV2 cells post-siRNA *GRN* knockdown, with/without 1 μg/ml C1q for 24 h; DAPI nuclei. Scale, 10 µm. **F**, **G** Percentage of BODIPY^+^ cells (**F**) and average lipid droplet size (**G**) in condition (**E**), 50 + cells/condition, 3 experiments; mean ± SEM, **p < 0.01, ****p < 0.0001 (Student’s* t* test)

We then investigated whether *GRN*–LOF iMGs could affect lipid droplet biogenesis since lysosomal acidification dysfunction with enlarged lysosomes in microglia could affect lipid metabolism such as lipid droplet biogenesis [44]. We stained iMGs with a fluorescent dye BODIPY to illuminate intracellular neutral lipid stores known as lipid droplets. *GRN*–LOF iMGs showed significantly greater lipid droplet content based on the number of lipid droplet (BODIPY^+^) cells and BODIPY fluorescence intensity than control cells (Fig. 5A–C). In addition, we observed a significant upregulation of perlipin3 (*PLIN3*) mRNA level in iMGs from FTD–*GRN* patients compared to control iMGs (Fig. 5D). *PLIN3* is a protein that coats intracellular lipid droplets. These data suggest that loss of *GRN* can cause lysosomal abnormalities and lipid dysregulation in human microglia from FTD–*GRN* patients, strengthening the role of PGRN critical for maintaining lysosomal homeostasis.

Lysosomal pathways are critical in processing and sorting exogenous and endogenous lipids [45]. Immune cells can accumulate lipid droplets in response to inflammatory conditions [46]. To investigate whether complement C1q is sufficient to induce lipid droplet formation in microglia and whether it is associated with *GRN* loss, BV2 microglial cells were transfected with GRN siRNA and treated with human complement C1q for 24 h. Lipid droplet formation was analyzed. Consequently, increased lipid droplet formation in BV2 microglial cells due to complement C1q was confirmed. It was found that in *GRN* KD condition, treatment with complement C1q induced markedly large sizes of lipid droplets (Fig. 5E–G). These results illustrate that human microglial model harboring *GRN*–LOF variants show immune dysfunction by excessive inflammation such as complement C1q activation, thus mediating abnormal lipid droplet accumulation. Furthermore, in *GRN*–LOF microglia, overactivation of inflammatory genes and complement could lead to defective phagocytosis and neurotoxic properties, which might increase lipid droplet production by reflecting lysosomal abnormalities.

## Discussion

Microglia are innate immune cells of the central nervous system. As major components of neuroinflammation, they have recently emerged as targets for neurodegenerative diseases [47]. PGRN is highly expressed in microglia. It is activated in reactive states due to injuries, aging, or disease pathology [9, 48]. FTD–*GRN* patients present increased disease-associated reactive microglia, pro-inflammatory cytokines, and microglial dystrophy [49]. However, whether human microglial functional defects caused by FTD-linked *GRN*–LOF variant directly contribute to FTD pathogenesis remains unclear. The current study investigated functional and pathological properties of microglia associated with PGRN haploinsufficiency by utilizing monocyte-derived iMGs from two FTD–*GRN* patients, one with a recurrent pathogenic variant and the other with a novel, likely pathogenic variant. Here, we demonstrated that LOF variants of *GRN* in the human monocyte-derived microglia-like cell model caused microglial dysfunction with abnormal TDP-43 aggregation induced by inflammatory milieu as well as impaired lysosomal function, thus representing an exacerbated disease phenotype. In contrast to the findings in patients with *GRN* variants, control subjects did not demonstrate PGRN-associated biomarkers or cell abnormalities. This contrast further substantiates the genetic integrity of our control group, affirming their absence of *GRN*-related abnormalities and underscoring the significance of the genetic differences observed in the study. Age-matched control subjects have been confirmed not to carry pathogenic variants of the *GRN* gene. Furthermore, these individuals did not exhibit any diseases associated with *GRN* abnormalities, nor was there any family history of such conditions reported. Microglial TDP-43 alterations were presumably a compositive phenotype reflecting exaggerated immune responses by activated complement and lysosomal abnormalities in PGRN-deficient microglia. Functional impairments in microglia due to *GRN* variants associated with FTD appear to be essential pathophysiological mechanisms underlying FTD–*GRN*.

iMGs from patients with FTD–*GRN* exhibited a reduction in phagocytic function compared to control iMGs, indicating a pro-inflammatory state. This observation aligns with broader findings that show alterations in morphology, cytokine production, secretion, and phagocytic ability in microglia when they are aberrantly activated or in a pro-inflammatory or diseased state [50]. Chronic pro-inflammatory conditions or pathological aberrant activation, particularly prevalent in neurodegenerative diseases, lead to a shift in microglial behavior characterized by impaired phagocytosis [51]. Consequently, we can assume that PGRN deficiency likely hinders their ability to maintain homeostatic molecular signatures and impairs their phagocytic capacity, further exacerbating neuroinflammation.

Accumulation of TDP-43 aggregates in the central nervous system is a common feature of many neurodegenerative diseases, such as amyotrophic lateral sclerosis (ALS), FTD, Alzheimer’s disease, and limbic-predominant age-related TDP-43 encephalopathy with dementia [52, 53]. Hereditary FTD–*GRN* shows ubiquitin-positive inclusions composed of TDP-43 in neuron and glial cells [3, 8]. Although aggregated TDP-43 has not been defined in microglia, this study demonstrates cytoplasmic TDP-43 positive inclusions in FTD–*GRN* patient-derived iMGs cultures under basal conditions without additional stressors. Microglial TDP-43 aggregates showed phosphorated TDP-43 (S409/410) and colocalization with ubiquitin protein. In addition, we identified elevated levels of TDP-43 and pathological TDP-43 (S409/410) in the insoluble fraction of FTD–*GRN* patient-derived iMGs by western blots (Fig. 3). To support this result, whether identical FTD–*GRN* patient-derived fibroblasts showed TDP-43 pathology comparable to those in FTD–*GRN* patient-derived iMGs was investigated. *GRN* patient-derived fibroblasts showed an increase in pTDP-43 (Ser409/410) immunoreactivity within the cytoplasm (Additional file 1: Fig. S3A). Furthermore, the expression of pTDP-43 (Ser409/410) in insoluble fractions of identical patient-derived fibroblasts carrying *GRN* variants and increased levels of TDP-43, ubiquitinated proteins, and p62 may support microglial TDP-43 proteinopathy (Additional file 1: Fig. S3B, C).

In a study using TDP-43-depleted BV2 microglial cells subjected to *GRN* knockdown, there was a notable accumulation of TDP-43 in the cytoplasm (Fig. 3F). This finding indicates a disposition for TDP-43, usually located in the nucleus of microglial cells, to aggregate in the cytoplasm when PGRN is deficient. In addition, recent research has shown that in the postmortem brains of patients with motor neuron disease with TDP-43 pathology, phosphorylated TDP-43 aggregates were present in the Iba1-positive microglial cells [54]. While TDP-43 is known to spread in a prion-like manner, moving from cell to cell in a seed-dependent and self-templating process [55], it appears feasible that cytoplasmic TDP-43 accumulation might be initiated by PGRN haploinsufficiency in a microglial environment where TDP-43 is typically confined to the nucleus under normal conditions.

Several studies have shown that inflammatory stimuli can promote TDP-43 aggregation and cytoplasmic mislocalization in microglial cells [20, 56–58]. In this study, we found several remarkable microglial phenotypes caused by PGRN haploinsufficiency in human microglia, which could not maintain homeostasis. They transformed into an inflammatory state mainly characterized by pro-inflammatory cytokine and complement activation with impaired phagocytosis, finally inducing exaggerated immune responses. The complement system is a rapid and efficient immune surveillance system. Its imbalance can contribute to various immune, inflammatory, and neurodegenerative diseases [59]. Upregulation of C1q and C3b is not only present in *GRN* mutation carriers, but also in genetically unexplained FTLD–TDP subtype A patients [60, 61]. Therefore, this study focused on complement activation in microglia from *GRN* variants to define the linkage between the complement system and TDP-43 proteinopathy. We found that direct complement C1q treatment in BV2 microglial cells triggered abnormal cytoplasmic aggregation of microglial TDP-43. In addition, complement C1q treatment with *GRN* loss condition markedly increased cytoplasmic aggregation of microglial TDP-43. Apart from the possibility that innate immune activation of microglial cells may exacerbate neuronal TDP-43 proteinopathy through the release of inflammatory cytokines, it was noteworthy that treatment with complement C1q in microglial cells self-triggered abnormal cytoplasmic aggregation of microglial TDP-43. TDP-43 is shuttled from the nucleus to the cytoplasm. It transiently forms cytoplasmic condensates through phase separation. This process can also lead to irreversible formation of permanent aggregates and fibrils in neurodegenerative diseases [62–64]. Recently, cytoplasmic TDP-43 mislocalization in monocyte-derived microglia-like cells of patients with ALS [65] and in lymphoblasts of patients with FTD–*GRN* [66] has been reported. In addition, *GRN*-deficient microglia exhibit extranuclear TDP-43 condensates with lipid droplets in a zebrafish model of traumatic brain injury [58]. Granulins have been shown to exacerbate TDP-43 toxicity in vivo in *Caenorhabditis elegans* and mice [67] and to alter the solubility of TPD-43, thereby modulating its phase separation and aggregation properties [68, 69]. The cell model of iMGs, similar to lymphocytes, may reflect the inflammatory state of FTD–*GRN,* including complement activation, inflammation, and other aging factors, which could result in cytoplasmic TDP-43 accumulation. Therefore, formation of cytoplasmic TDP-43 aggregates by complement activation suggests that *GRN*–LOF microglia are sufficient to trigger the pathological process of FTD–*GRN*.

Due to inflammation induced by PGRN haploinsufficiency, upregulated cell death might obscure TDP-43 accumulation in human brain microglia with FTD–*GRN*. Investigating this, we utilized cleaved capsace-3 as a marker of apoptosis to access cytotoxicity. As a result, we found that iMGs from patients with FTD–*GRN* showed significantly increased cleaved caspase-3 positive immunoreactivity compared to control iMGs (Additional file 1: Fig. S4). Furthermore, we examined endolysosomal membrane permeabilization of iMGs from patients with FTD–*GRN* and control iMGs, hypothesizing that impairment of lysosomal membrane integrity would cause lysosomal-dependent cell death [70]. Utilizing immunofluorescence staining techniques, we examined the cellular distribution of galectin-3 (Gal-3). Gal-3 is a cytosolic protein known to localize to damaged lysosomes, functioning as a sensitive marker for lysosomal leakage [71]. Our observations revealed that in iMGs obtained from patients with FTD–*GRN*, a substantial proportion of Gal-3 was present as punctate formations. These formations indicate intracellular vesicle rupture, commonly triggered by amyloid proteins such as α-synuclein, tau, and mutant Huntingtin [72]. In contrast, such punctate formations of Gal-3 were scarcely observed in control microglia, highlighting a distinct pattern in FTD–*GRN* patient-derived cells (Additional file 1: Fig. S5). The observations indicate potential challenges in detecting TDP-43 accumulation in human brain microglia, which might be attributed to activated cell death, potentially influenced by diminished lysosomal membrane integrity.

PGRN is an intracellular and extracellular precursor protein that undergoes proteolytic cleavage, forming individual granulin peptides [73]. In the context of PGRN cleavage, it is noteworthy that this process yields paragranulin, with an approximate molecular weight of 3.5 kDa, and granulins A–G, each approximately 7 kDa in size [73, 74]. Recent reports have suggested that granulin peptides may be critical in generating TDP-43 toxicity in FTD–*GRN* [67]. Notably, despite the reduction of its precursor PGRN due to haploinsufficiency, granulin F levels have been found to be increased in regions of the human FTD–*GRN* brain [74]. Despite the controversy surrounding the diverse functions and variable expression of individual granulin peptides in pathological states, emerging evidence that links granulin peptides to prion-like TDP-43 cytoplasmic inclusions supports the hypothesis of their potential pathognomonic role in FTD–*GRN* [68, 75, 76].

PGRN is a critical lysosomal chaperone required for lysosomal function and the ability of microglia to counteract misfolded proteins [7]. It has been reported that PGRN deficiency is linked to lysosomal dysfunction, which can influence lysosomal acidification and enzymatic activity, defective autophagy, and lipofuscinosis [77, 78]. Our findings are consistent with recent studies showing that PGRN protein is expressed in lysosomes of human microglial cells. FTD–*GRN* patient-derived iMGs reveal lysosomal abnormalities, including enlarged lysosomes, alteration of lysosomal genes, abnormal lipid droplet accumulation, and TFEB activation. Activation of TFE3/TFEB has been shown to drive expression of inflammation genes [79]. These data suggest that lysosome abnormalities in microglia can result in a feedback loop through activation of the TFEB pathway, which could drive the expression of inflammatory genes and the activation of target genes by lysosomal damage. PGRN levels have been linked to expression of several genes, including *SORT1* and *TMEM106B* in lysosomes [42, 80]. Induced lysosome dysfunction caused by increased expression of TMEM106B can inhibit the processing of PGRN into granulins [81]. Consistent with previous studies, we found that iMGs from FTD–*GRN* patients resulted in lysosomal enlargement and dysregulated markers of lysosomes along with increased lysosomal protein levels such as SORT1 and TMEM106B. Overexpression of TMEM106B can cause translocation of TFEB to the nucleus and induces upregulation of coordinated lysosomal expression and regulation network [82]. The present study demonstrated a significant elevation in the relative mRNA expression of *LGALS3*, encoding Gal-3, in iMGs from patients with FTD–*GRN*. Furthermore, the occurrence of Gal-3 and its puncta formations, which are absent in control iMGs, were evident in the iMGs derived from patients with FTD–*GRN* (Additional file 1: Fig. S5). Our results align with existing data indicating an upregulation of Gal-3 in both patients with FTD–*GRN* and *Grn* LoF mice [83, 84]. These observations, coupled with our previous findings of lysosomal membrane permeabilization triggered by PGRN deficiency in human iPSC-derived *GRN−*/*−* microglia, strongly suggest the occurrence of lysosomal damage in iMGs associated with FTD–*GRN*.

Furthermore, we examined the co-localization of lipid droplets (BODIPY) with lysosomes immunostained with LAMP1 to investigate the lipophagic delivery of lipid droplets to lysosomes. Lipid droplets in control-derived iMGs clearly showed co-localization with lysosomes, whereas partial co-localization of lipid droplets with lysosomes was present in iMGs from patients with FTD–*GRN* (Additional file 1: Fig. S6). The current results demonstrate that PGRN haploinsufficiency-induced abnormal lipid droplets in microglia may interfere with lipid degradation in microglial lysosomes.

In addition, TDP-43 pathology may disrupt lysosomal function, driving further pathology. Loss of nuclear TDP-43 is a key aspect of TDP-43 pathology that may disrupt the autophagy–lysosomal and endolysosomal systems [85–89]. Therefore, defective microglial lysosome by PGRN loss might lead to impaired phagocytic and autophagic clearance of cellular waste and debris as well as toxic protein aggregates. Conversely, TDP-43 aggregation might be further exacerbated by lysosomal abnormalities in PGRN-deficient microglia.

It has been reported that inflammatory and metabolic changes in immune cells involved in upregulated fatty acid production can cause formation of lipid droplets [46]. Accumulation of lipid droplets in microglia is known to represent a dysfunctional and pro-inflammatory state in the aging brain [90, 91]. *GRN* knockout by gene editing can promote lipid droplet accumulation in microglia, resulting in phagocytic dysfunction and activation of pro-inflammatory responses [90]. This study revealed that FTD–*GRN* patient-derived iMGs could induce lipid-droplet formation accompanied by activation of inflammatory cytokines, including complement. Furthermore, direct complement C1q treatment induced lipid droplet formation in BV2 microglial cells. Additional *GRN* loss increased lipid droplet sizes. These results suggest that an impaired lysis mechanism caused by lysosomal abnormalities can lead to the excessive accumulation of lipid droplets with activated inflammatory conditions in FTD–*GRN* patient-derived iMGs.

Recent studies have aimed at a therapeutic approach for FTD–*GRN* to restore CNS PGRN levels [92] using adenovirus-associated virus-based gene therapy, SORT1-binding antibodies, and small molecules modulators (such as suberoylanilide hydroxamic acid, methyltransferase inhibitors, nor-binaltorphimine dihydrochloride, and dibutyryl-cAMP, sodium salt [93–96]. Despite encouraging success in preclinical studies, a barrier remains due to the lack of suitable human and mouse models for therapeutic development of FTD–*GRN*. Mice with heterozygous *Grn* deletions do not exhibit behavioral or neuropathological changes typical of *GRN* heterozygosity in humans [97, 98]. Recent approaches using induced pluripotent stem cell-derived microglia are now available. However, relative complexity, high variability, and extended timeframe are required to generate cell models. Moreover, iPSC-derived microglia may not accurately contain the heterogeneity of clinical features observed in the disease process by pathogenic variants due to loss of epigenetic factors during reprogramming [99]. In this study, we used an iMGs model derived from human monocytes, a rapid and minimally invasive system that allows for multiple sampling at various stages of the disease. This cell model can recapitulate changes in microglia during disease progression. Such changes can be correlated with clinical data (brain imaging and clinical disease progression), which may bridge the gap between clinical studies by providing a better clinical outcome [25, 30, 65]. Therefore, iMGs could be used as an in vitro platform method or a preclinical study tool to analyze their functional defects through genetic mutations and to evaluate therapeutic drugs.

However, this study has some limitations. First, it was not possible to enroll various types of patients diagnosed with FTD–*GRN*. In contrast to the Caucasian population, the Asian population revealed a significantly lower frequency of FTD–*GRN* [100–105]. Furthermore, differences in clinical severity of the disease, patients’ states, other genetic modifiers, and sex-based microglial effects might have affected results since this study only investigated two types of patient-derived iMGs models. Additional patients with different FTD–*GRN* pathogenic variants might provide more valuable experimental results. Second, microglial TDP-43 aggregates in FTLD–*GRN* human brain tissues have not been reported yet. However, biochemical TDP-43 phenotypes closely resemble those observed in neuron. Given that the possibility of TDP-43 aggregation in microglial cells has recently been reported, further studies are needed to confirm the formation of TDP-43 aggregates according to different phenotypic markers of microglial cells in patient tissues. Third, we could not analyze the effects of individual granulin peptides produced through the proteolytic cleavage of PGRN. Specific granulin peptides have been implicated in liquid–liquid phase separation associated with TDP-43 accumulation [68]. However, our research did not extend to investigating the impact of individual granulin peptides on TDP-43. Given the conflicting results emerging from various studies regarding individual granulin peptides, there is a clear need to develop antibodies that can specifically detect these peptides and further research into their interactions [74–76]. Lastly, although this study focused only on the role of PGRN in human microglia, its effect on interactions with various types of neuronal cells in the brain environment was not determined. Therefore, further studies are necessary to elucidate the impact of PGRN, such as utilization of 3D modeling that incorporates a brain microenvironment with different neuronal cell types, embodying the complexity of a brain’s homeostatic and diseased states.

## Conclusions

Overall, our study supports the identification of several pathological phenotypes and functional impairments of PGRN haploinsufficiency microglia, including hyperinflammation due to microglial activation, defective phagocytosis, lipid droplet accumulation, and lysosomal abnormalities using FTD–*GRN* patient-derived microglia. This study provides a novel finding of cytoplasmic TDP-43 accumulation in microglia, which has not been previously observed. Excessive inflammation and lysosomal abnormalities in microglia due to PGRN haploinsufficiency might be sufficient to cause cytoplasmic TDP-43 aggregates. Our results suggest that microglia characterization of PGRN haploinsufficiency will provide further insight into neuropathological phenotypes and better define mechanisms underlying FTD–*GRN*. In addition, the iMGs model has potential to be used to assess preclinical efficacy of new therapies targeting relevant LOF variants that contribute to FTD–*GRN*.

### Supplementary Information

**Additional file 1: Materials and Methods S1.** For immunostaining, the following primary antibodies were used in this study: Galectin-3 (Gal-3, 1:100, Proteintech Cat# 14979–1-AP, RRID: AB_2136768) and cleaved-caspase3 (1:100, Cell Signaling, Cat# #9661S, RRID: AB_2910623). For determining the number of galectin-3 puncta per cell in the microscopy images were quantified using the software ImageJ. For colocalization analysis of LAMP1 and BOPIDY, Fiji software of ImageJ was used for colocalization analysis. The regions of interest (ROIs) were created with the circular enlargement of local fluorescence intensity maxima or lasso tool. The overlap and direct apposition of segmented ROIs were collectively defined as an association. The overlap was defined as colocalization. To quantify apoptosis, cleaved-caspase-3 positive cells were counted using the counter plugin and divided by the number of DAPI + nuclei in each field. **Figure S1.** Family members who underwent DNA analysis for segregation data are indicated with V/W for a heterozygous variant and W/W for a homozygous reference allele. Solid black denotes patients affected with FTD. A black-shaded pattern indicates patient presenting with only dementia symptoms without evidence of FTD. A single midline indicates an individual carrying a variant without clinical symptoms at the time of family pedigree generation. **C** Proband and his older son carried the same variant affecting the initiation codon (c.1A > G), while other family members did not present with variants. **Figure S2.** **A, C** Images of the patient carrying an impaired *GRN* initiation codon presenting with asymmetric cortical atrophy of the left cerebral hemisphere in MRI. FDG–PET images demonstrate metabolic impairment in the left parieto-temporal cortices, medial frontal cortex, and right parietal cortex. **B, D, and E.** Brain MRI of the patient with a premature stop codon in *GRN* revealed mild atrophy of left frontal and temporal lobes. The left anterolateral temporal cortex showed a prominent decrease in metabolic activity. The left fronto-temporo-parietal cortex showed diffuse metabolic impairment. However, amyloid deposits were absent in both lateral temporal, frontal, posterior cingulate, and parietal cortices. **Figure S3. A** Representative fluorescence images of phosphorylated TDP-43 at Ser409/410 (pTDP-43, green) in FTD–*GRN* patients-derived fibroblast and control-derived fibroblast. Nuclei were stained with DAPI. Scale bar, 10 µm. **B** Western blot of TDP-43, pTDP-43, ubiquitin, and p62 in soluble and insoluble fractions from FTD–*GRN* patients-derived fibroblast and control-derived fibroblast. GAPDH was used as a loading control in soluble fractions. **C** Quantification of normalized TDP-43, pTDP-43, ubiquitination, p62, and PGRN expression in soluble and insoluble fractions (n = 3). Values are presented as mean ± SEM. Comparisons were made against control-derived fibroblasts (**p < 0.01, ***p < 0.001, ****p < 0.0001; one-way ANOVA with post hoc Tukey’s test). **Figure S4. A** Representative fluorescence images of IBA1 (green) and cleaved-caspase 3 (red) to identify dead cells in FTD–*GRN* patients-derived iMGs and control-derived iMGs. Nuclei were stained with DAPI. Scale bar, 10 µm. **B** Average cell death rates of *GRN* mutations were measured as the percentage of cleaved-caspase 3-positive cells in IBA1-positive cells. Over 30 cells were quantified per experiment from three biologically independent experiments. Values are presented as mean ± SEM. Comparisons were made against control-derived iMGs (****p < 0.0001; one-way ANOVA with post hoc Tukey’s test). **Figure S5. A** Representative fluorescence images of Galectin-3 (Gal-3, red) in FTD–*GRN* patients-derived iMGs and control-derived iMGs. Nuclei were stained with DAPI. Scale bar, 10 µm. **B** Quantification of the percentage of galectin-3 puncta in (**A**). Galectin-3 puncta is the marker of vesicle rupture. Over 30 cells were quantified per experiment from three biologically independent experiments. Values are presented as mean ± SEM. Comparisons were made against control-derived iMGs (****p < 0.0001; one-way ANOVA with post hoc Tukey’s test. **Figure S6. A** Representative fluorescence images of BODIPY (lipid droplets, green) and LAMP1 (lysosome, red) in FTD–*GRN* patients-derived iMGs and control-derived iMGs. The right panel shows higher magnification views of white box regions. Nuclei were stained with DAPI. Scale bar, 10 µm. **B** Quantification of colocalization between LAMP1 and BODIPY in (**A**). Over 30 cells were quantified per experiment from three biologically independent experiments. Values are presented as mean ± SEM. Comparisons were made against control-derived iMGs (****p < 0.0001; one-way ANOVA with post hoc Tukey’s test). **Figure S7.** full uncropped Gels and Blots images. **Table S1.** Clinical characteristics of patients with FTD–*GRN* and controls.

## Data Availability

The IRB of Hanyang University Hospital reviewed all requests for raw and analyzed data and related materials to determine whether each request was subjected to any intellectual property or confidentiality restrictions. Data that support results of this study can be obtained from the corresponding authors upon reasonable request.

## Abbreviations

ALS: Amyotrophic lateral sclerosis
FTLD: Frontotemporal lobar degeneration
FTD–*GRN*: *Granulin*-Linked frontotemporal dementia
FTD: Frontotemporal dementia
GRN: Progranulin gene
iMGs: Induced microglia-like cells
LAMP1: Lysosomal-associated membrane protein 1
LOF: Loss of function
nfavPPA: Nonfluent/agrammatic variant primary progressive aphasia
NfL: Neurofilament light chain
PGRN: Progranulin
PLIN3: Perlipin3
SORT1: Sortilin
svPPA: Semantic variant primary progressive aphasia
TDP-43: TAR DNA-binding protein 43
TFEB: Transcription factor EB
TMEM106B: Transmembrane protein 106 B
TREM2: Triggering receptor expressed on myeloid cells 2
