# Supplementary material for: Progranulin haploinsufficiency mediates cytoplasmic TDP-43 aggregation with lysosomal abnormalities in human microglia
Source: J Neuroinflammation. 2024 Feb 13;21:47. doi: 10.1186/s12974-024-03039-1 (PMC10863104; doi:10.1186/s12974-024-03039-1)
Supplement: Supplementary file 1 — Additional file 1: Materials and Methods S1. For immunostaining, the following primary antibodies were used in this study: Galectin-3 (Gal-3, 1:100, Proteintech Cat# 14979–1-AP, RRID: AB_2136768) and cleaved-caspase3 (1:100, Cell Signaling, Cat# #9661S, RRID: AB_2910623). For determining the number of galectin-3 puncta per cell in the microscopy images were quantified using the software ImageJ. For colocalization analysis of LAMP1 and BOPIDY, Fiji software of ImageJ was used for colocalization analysis. The regions of interest (ROIs) were created with the circular enlargement of local fluorescence intensity maxima or lasso tool. The overlap and direct apposition of segmented ROIs were collectively defined as an association. The overlap was defined as colocalization. To quantify apoptosis, cleaved-caspase-3 positive cells were counted using the counter plugin and divided by the number of DAPI + nuclei in each field. Figure S1. Family members who underwent DNA analysis for segregation data are indicated with V/W for a heterozygous variant and W/W for a homozygous reference allele. Solid black denotes patients affected with FTD. A black-shaded pattern indicates patient presenting with only dementia symptoms without evidence of FTD. A single midline indicates an individual carrying a variant without clinical symptoms at the time of family pedigree generation. C Proband and his older son carried the same variant affecting the initiation codon (c.1A > G), while other family members did not present with variants. Figure S2. A, C Images of the patient carrying an impaired GRN initiation codon presenting with asymmetric cortical atrophy of the left cerebral hemisphere in MRI. FDG–PET images demonstrate metabolic impairment in the left parieto-temporal cortices, medial frontal cortex, and right parietal cortex. B, D, and E. Brain MRI of the patient with a premature stop codon in GRN revealed mild atrophy of left frontal and temporal lobes. The left anterolateral temporal cort [file 12974_2024_3039_MOESM1_ESM.docx]

**Additional Materials and Methods**

For immunostaining, the following primary antibodies were used in this study: Galectin-3 (Gal-3, 1:100, Proteintech Cat# 14979-1-AP, RRID: AB_2136768) and cleaved-caspase3 (1:100, Cell Signaling, Cat# #9661S, RRID: AB_2910623). For determining the number of galectin-3 puncta per cell in the microscopy images were quantified using the software ImageJ. For colocalization analysis of LAMP1 and BOPIDY, Fiji software of ImageJ was used for colocalization analysis. The regions of interest (ROIs) were created with the circular enlargement of local fluorescence intensity maxima or lasso tool. The overlap and direct apposition of segmented ROIs were collectively defined as an association. The overlap was defined as colocalization. To quantify apoptosis, cleaved-caspase-3 positive cells were counted using the counter plugin and divided by the number of DAPI+ nuclei in each field.


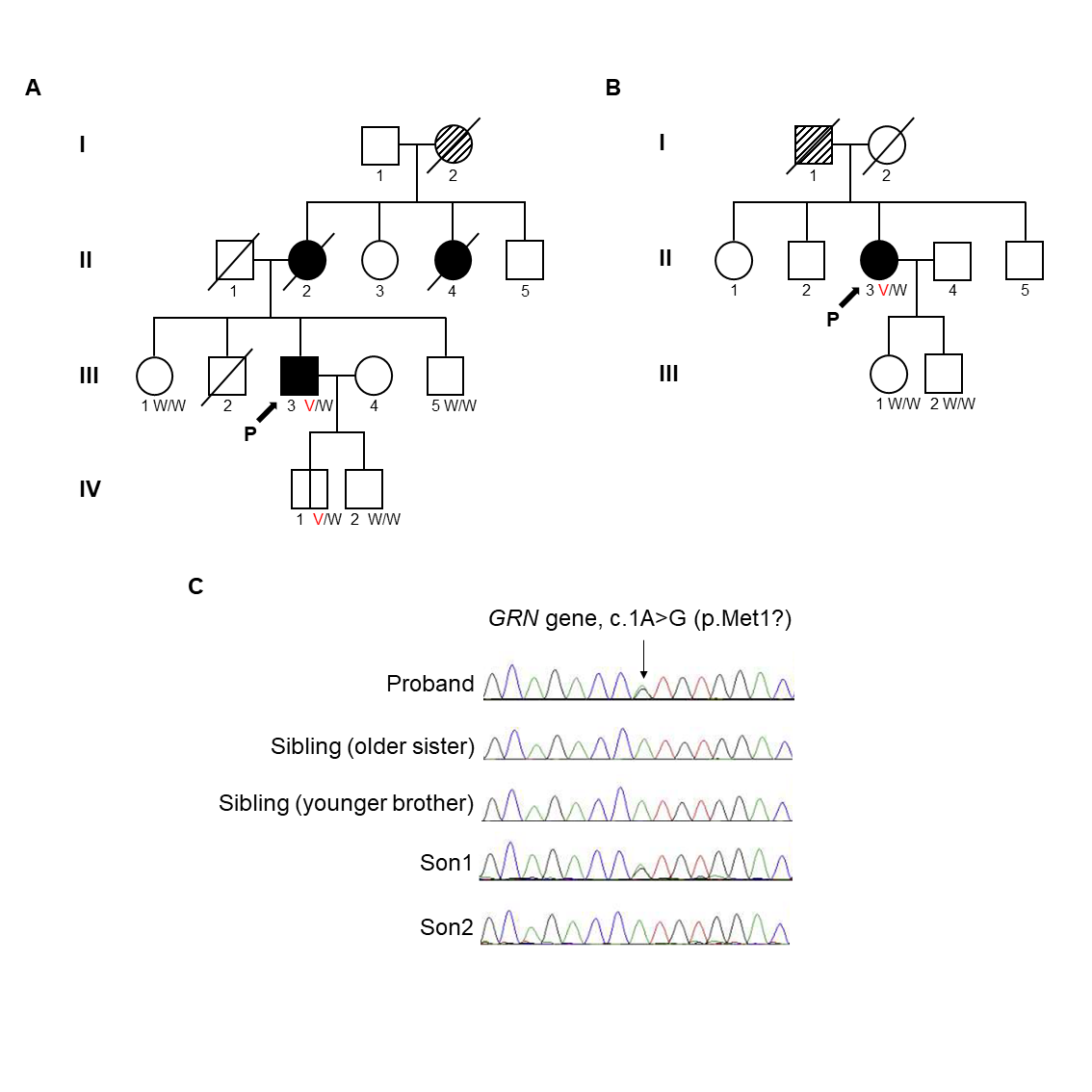
 **Figure S1.** **Family pedigrees of patient 1 carrying p.M1? variant of *GRN* (A) and patient 2 carrying p.W147* variant of *GRN* (B) and Sanger sequencing chromatograms of patient 1 family members (C).** Family members who underwent DNA analysis for segregation data are indicated with V/W for a heterozygous variant and W/W for a homozygous reference allele. Solid black denotes patients affected with FTD. A black-shaded pattern indicates patient presenting with only dementia symptoms without evidence of FTD. A single midline indicates an individual carrying a variant without clinical symptoms at the time of family pedigree generation. **C.** The proband and his older son carried the same variant affecting the initiation codon (c.1A>G), while other family members did not present with variants.


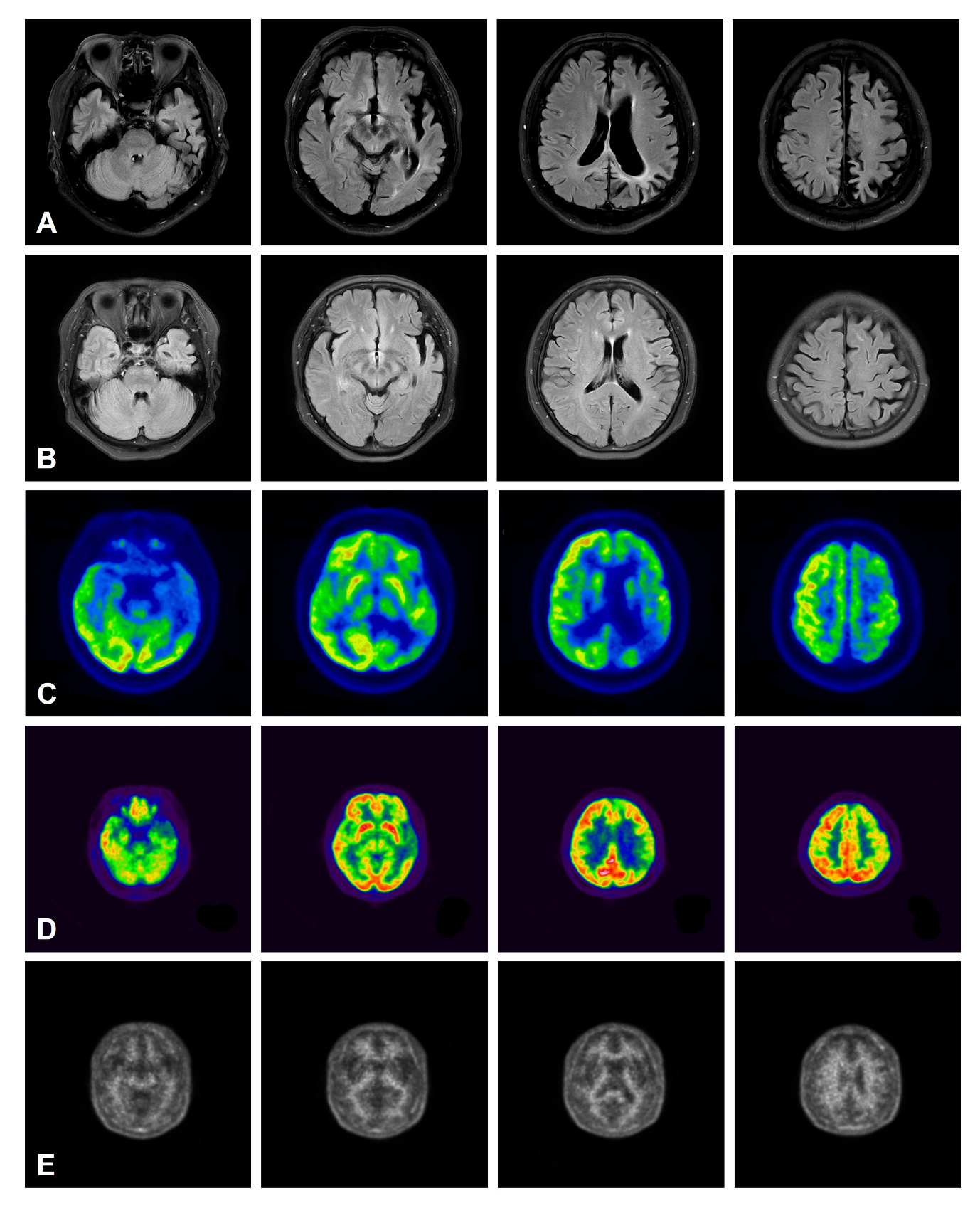
**Figure S2.** **T2-weighted fluid-attenuated inversion recovery axial MRI images, brain fluorine-18 fluorodeoxyglucose positron-emission tomography (FDG-PET) images, and amyloid positron-emission tomography (PET) images of patients 1 and 2.**

**A and C.** Images of the patient carrying an impaired *GRN* initiation codon presenting with asymmetric cortical atrophy of the left cerebral hemisphere in MRI. FDG-PET images demonstrate metabolic impairment in the left parieto-temporal cortices, medial frontal cortex, and right parietal cortex. **B, D, and E.** Brain MRI of the patient with a premature stop codon in *GRN* revealed mild atrophy of left frontal and temporal lobes. The left anterolateral temporal cortex showed a prominent decrease in metabolic activity. The left fronto-temporo-parietal cortex showed diffuse metabolic impairment. However, amyloid deposits were absent in both lateral temporal, frontal, posterior cingulate, and parietal cortices.


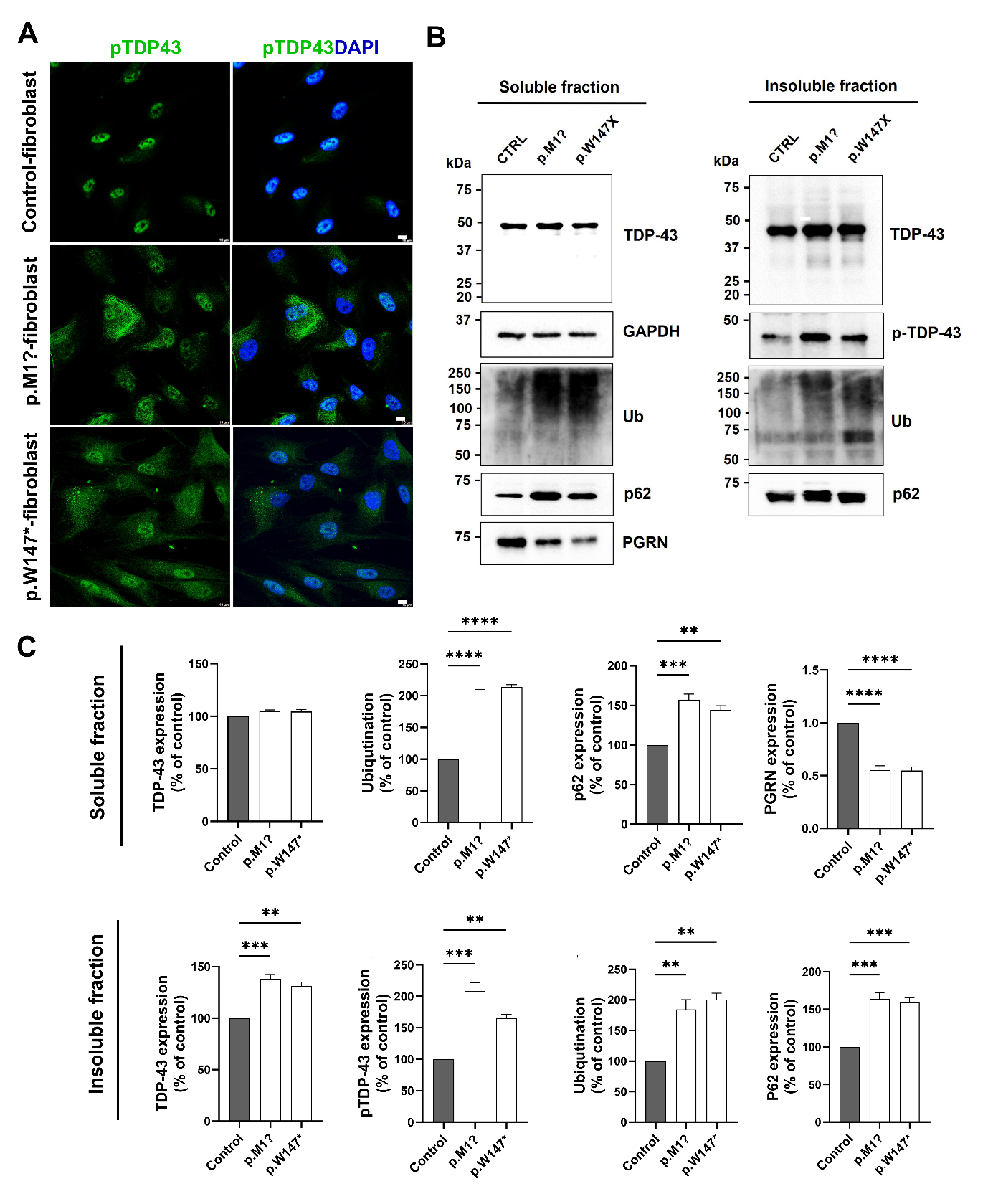


**Figure S3.** Patient-derived fibroblast carrying *GRN* mutations shows abnormal TDP-43 proteinopathy.

**A**. Representative fluorescence images of phosphorylated TDP-43 at Ser409/410 (pTDP-43, green) in FTD-*GRN* patients-derived fibroblast and control-derived fibroblast. Nuclei were stained with DAPI. Scale bar, 10 µm. **B.** Western blot of TDP-43, pTDP-43, ubiquitin, and p62 in soluble and insoluble fractions from FTD-*GRN* patients-derived fibroblast and control-derived fibroblast. GAPDH was used as a loading control in soluble fractions. **C.** Quantification of normalized TDP-43, pTDP-43, ubiquitination, p62, and PGRN expression in soluble and insoluble fractions (n = 3). Values are presented as mean ± SEM. Comparisons were made against control-derived fibroblasts (***P* < 0.01, ****P* < 0.001, *****P* < 0.0001; one-way ANOVA with post hoc Tukey’s test).


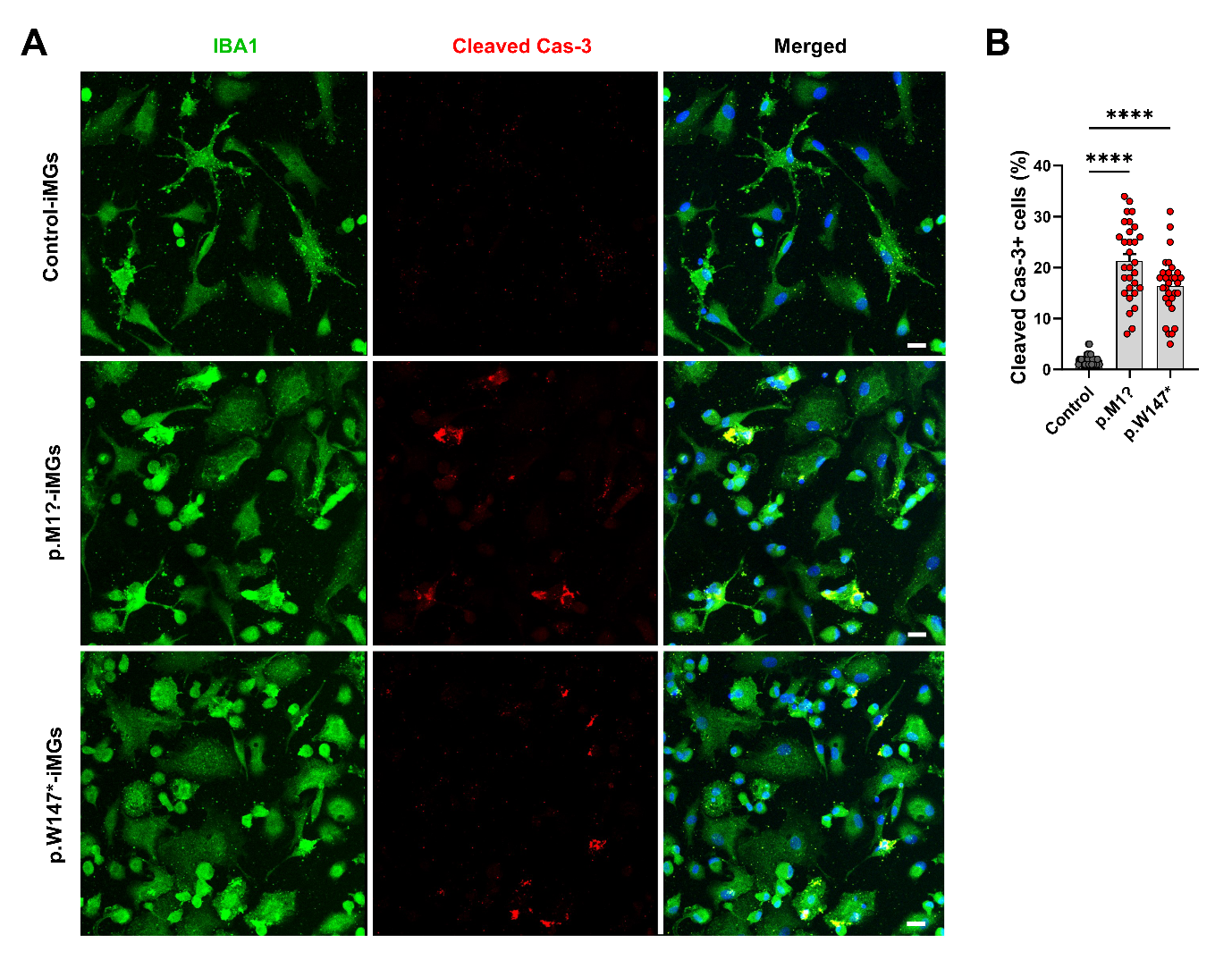


**Figure S4.** *PGRN* haploinsufficiency induced apoptosis in microglial cells.

**A**. Representative fluorescence images of IBA1 (green) and cleaved-caspase 3 (red) to identify dead cells in FTD-*GRN* patients-derived iMGs and control-derived iMGs. Nuclei were stained with DAPI. Scale bar, 10 µm. **B**. Average cell death rates of *GRN* mutations were measured as the percentage of cleaved-caspase 3-positive cells in IBA1-positive cells. Over 30 cells were quantified per experiment from three biologically independent experiments. Values are presented as mean ± SEM. Comparisons were made against control-derived iMGs (*****P* < 0.0001; one-way ANOVA with post hoc Tukey’s test).


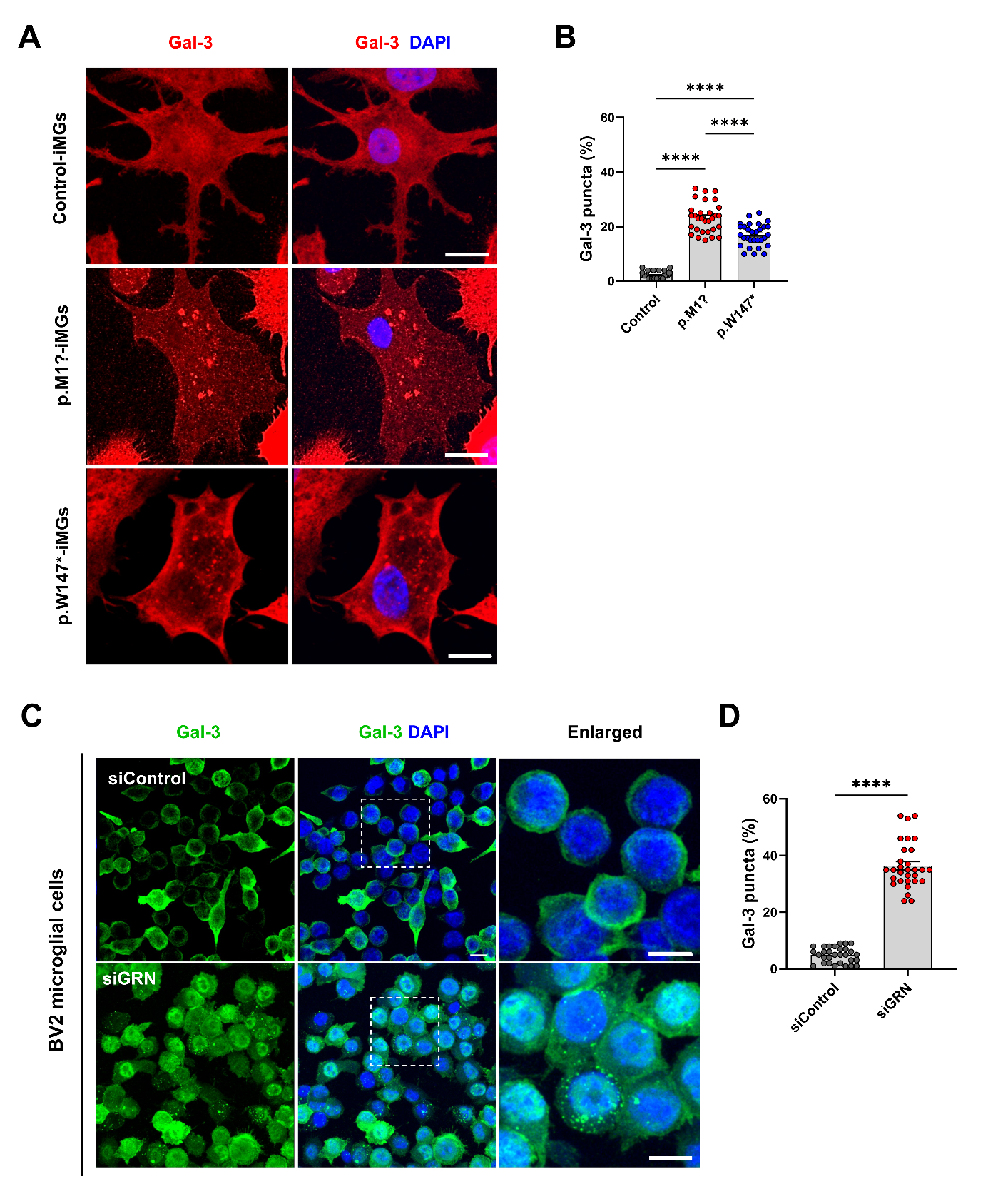


**Figure S5.** Patient-derived iMGs carrying *GRN* variants exhibit lysosomal damage.

**A**. Representative fluorescence images of Galectin-3 (Gal-3, red) in FTD-*GRN* patients-derived iMGs and control-derived iMGs. Nuclei were stained with DAPI. Scale bar, 10 µm. **B**. Quantification of the percentage of galectin-3 puncta in (**A**). Galectin-3 puncta is the marker of vesicle rupture. Over 30 cells were quantified per experiment from three biologically independent experiments. Values are presented as mean ± SEM. Comparisons were made against control-derived iMGs (*****P* < 0.0001; one-way ANOVA with post hoc Tukey’s test).


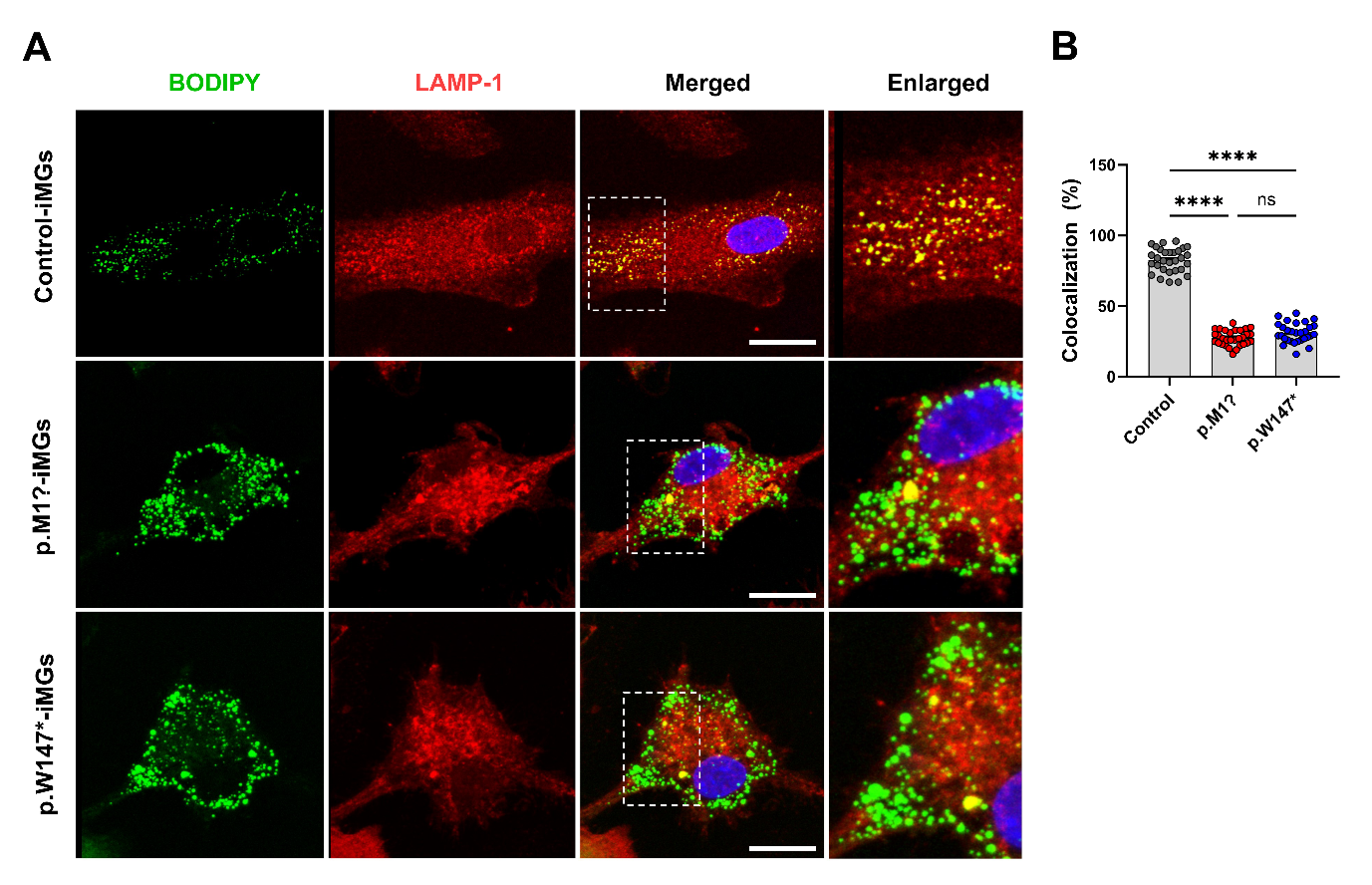


**Figure S6.** Patient-derived iMGs carrying *GRN* variants exhibit impaired lipophagy of lipid droplets.

**A**. Representative fluorescence images of BODIPY (lipid droplets, green) and LAMP1 (lysosome, red) in FTD-*GRN* patients-derived iMGs and control-derived iMGs. The right panel shows higher magnification views of white box regions. Nuclei were stained with DAPI. Scale bar, 10 µm. **B**. Quantification of colocalization between LAMP1 and BODIPY in (**A**). Over 30 cells were quantified per experiment from three biologically independent experiments. Values are presented as mean ± SEM. Comparisons were made against control-derived iMGs (*****P* < 0.0001; one-way ANOVA with post hoc Tukey’s test).


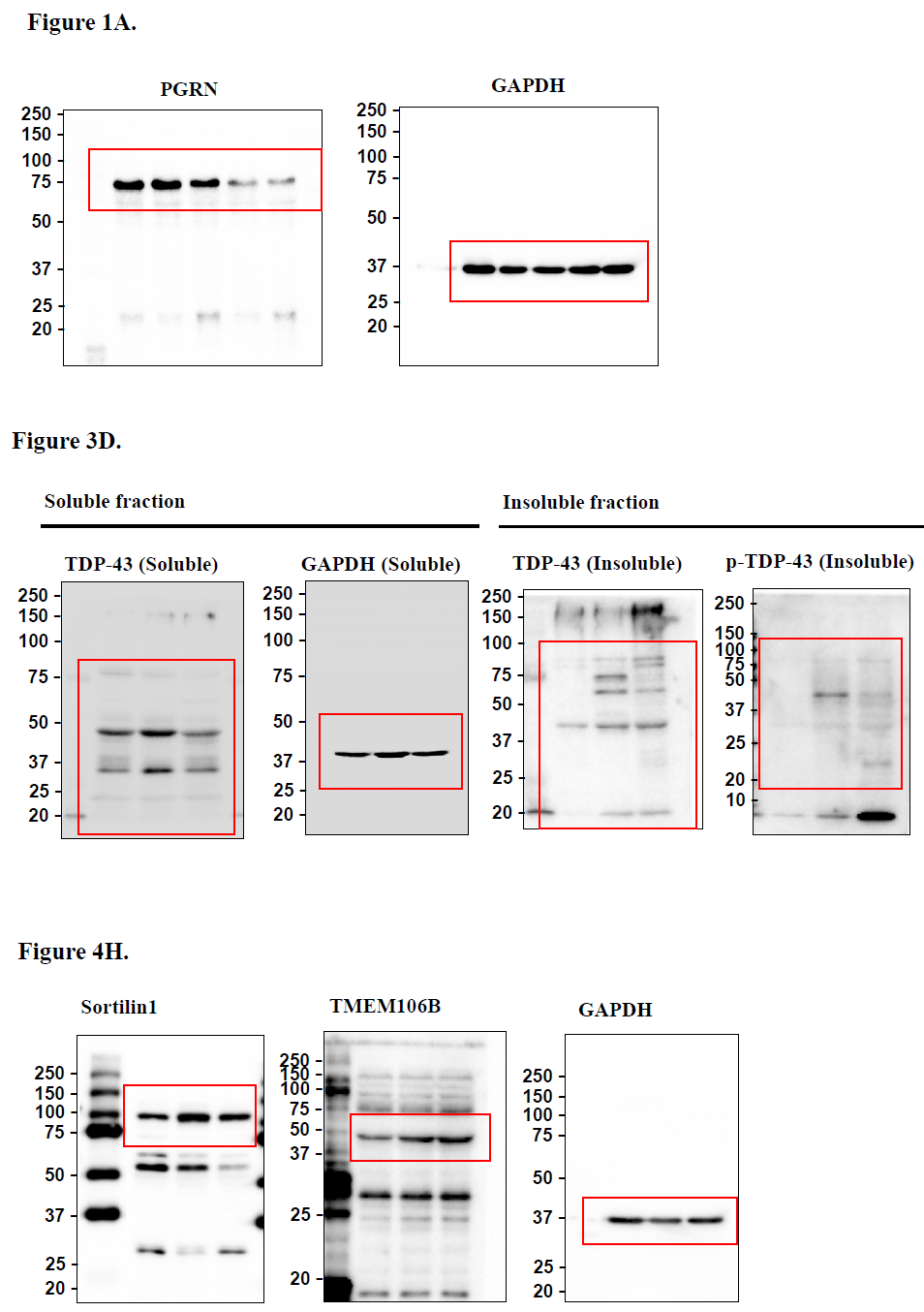


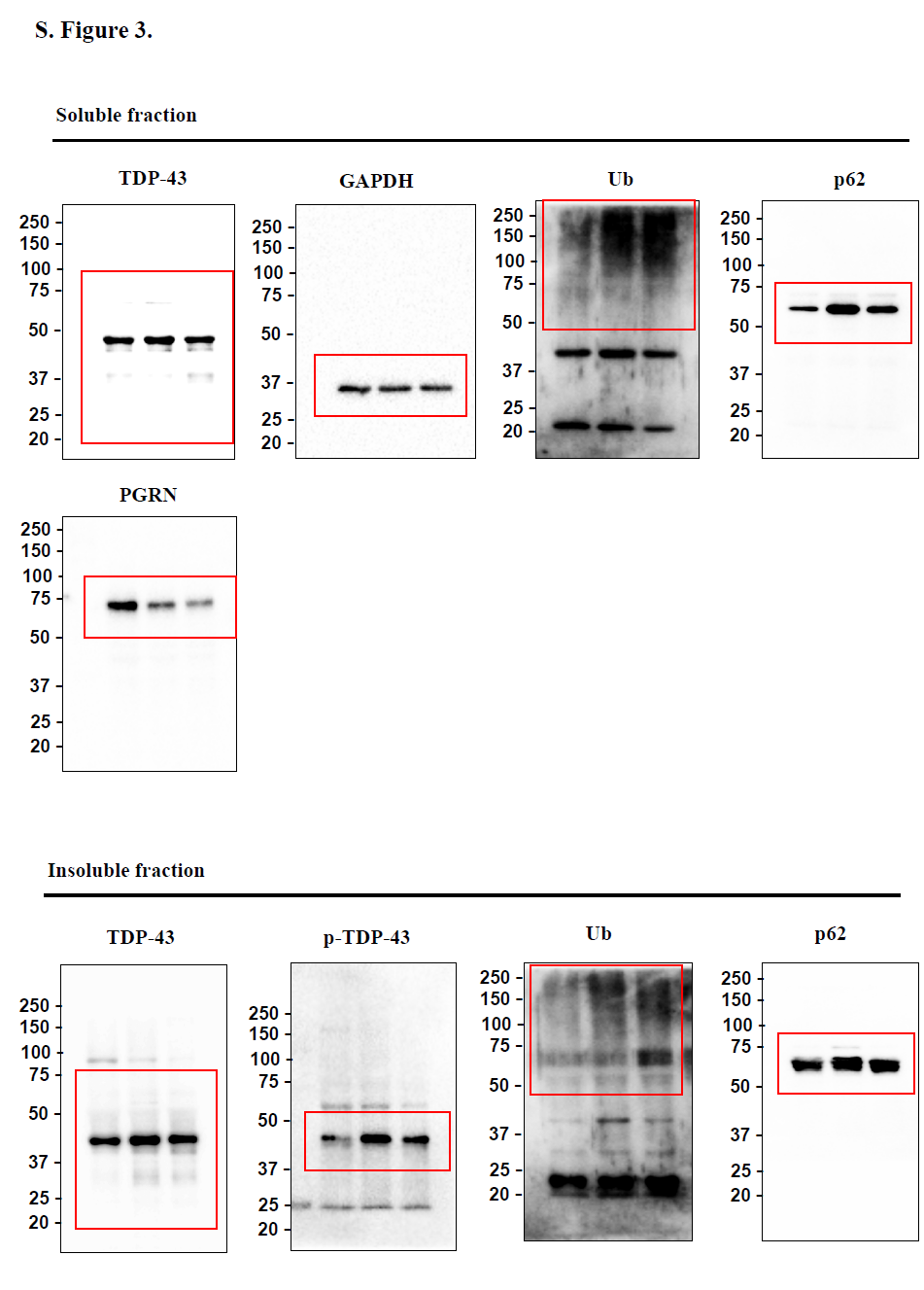


**Figure S7.** full uncropped Gels and Blots images.

**Table S1. Clinical characteristics of patients with FTD-*GRN* and controls**

| Characteristic | Amino  acid  change | Ag  e | S  e  x | Education  level, y | Age of  onset, y | Disease  duration  at first  evaluation, y | Main  disorders | Frontal lobe dysfunction | | Speech and language dysfunction | | | | | Parietal  syndrome | Amnestic syndrome |
| --- | --- | --- | --- | --- | --- | --- | --- | --- | --- | --- | --- | --- | --- | --- | --- | --- |
|  |  |  |  |  |  |  |  | Personality/ behavioural changes | Execution | Fluency  (Spontaneous  speech) | Confrontation  naming | Agrammatism | Written  sentence  comprehension | Repetition |  |  |
| FTD-GRN | p.M1? | 61 | M | 17 | 53 | 8 | Aphasia | + | + | + | + | - | + | - | + | + |
|  | p.W147* | 62 | F | 12 | 60 | 2 | Aphasia | - | - | - | + | - | - | - | - | - |
| Control-1 | N/A | 55 | M | N/A | N/A | N/A | N/A | N/A | N/A | N/A | N/A | N/A | N/A | N/A | N/A | N/A |
| Control-2 | N/A | 63 | F | N/A | N/A | N/A | N/A | N/A | N/A | N/A | N/A | N/A | N/A | N/A | N/A | N/A |
| Control-3 | N/A | 75 | M | N/A | N/A | N/A | N/A | N/A | N/A | N/A | N/A | N/A | N/A | N/A | N/A | N/A |

Abbreviations: +, prominent impairment; -, no deficit, y-years; N/A, not applicable.
